# Development of a shyness scale for Chinese high school students

**DOI:** 10.3389/fpsyg.2026.1895425

**Published:** 2026-10-22

**Authors:** Fan Huang, Ping Gao, Luzhuang Sun, Xingyu Zhang, Chaobing Hu

**Affiliations:** 1 Chongqing Business Vocational College, Chongqing, China; 2 School of Economics and Management, Chongqing University of Posts and Telecommunications, Chongqing, China; 3 Student Mental Health Education and Consultation Center, Chongqing Normal University, Chongqing, China; 4 School of Educational Sciences, Chongqing Normal University, Chongqing, China

**Keywords:** shyness, Chinese high school students, scale development, bifactor model, measurement invariance, multivariate generalizability theory, cross-validation

## Abstract

**Objective:**

Grounded in Chinese indigenous culture and the psychosocial developmental characteristics of high school adolescents, this study developed and validated a culturally adapted shyness scale for Chinese high school students—the High School Student Shyness Scale (HSSS). The instrument aims to provide a valid and localized psychometric tool for objective shyness assessment and empirical research on adolescent shyness.

**Methods:**

The preliminary dimensional framework and items were established through literature review, open-ended questionnaire investigation, and expert review. Participants were recruited from district and county-level high schools in Chongqing, China, covering both urban and rural household registration backgrounds, using a combination of cluster sampling and convenience sampling. The study was conducted in stages: an open-ended questionnaire survey was first conducted with 138 high school students to generate indigenous items; 487 valid samples were used for item analysis and exploratory factor analysis (EFA) to screen items and construct the initial factor structure; an independent cohort of 427 students was administered for confirmatory factor analysis (CFA), alternative model comparisons, measurement invariance tests, multivariate generalizability theory (MGT) analysis, and systematic reliability and validity assessment; an additional 176 students completed a four-week retest to evaluate test–retest reliability; and 200 participants completed the classic Cheek and Buss Shyness Scale to examine the criterion-related validity of the newly developed scale.

**Results:**

The final 18-item HSSS comprises three dimensions: Withdrawal from avoidant speech and behavior, Negative self-consciousness, and Negative emotional experience. EFA using Principal Axis Factoring with Promax rotation and parallel analysis supported a three-factor structure (KMO = 0.947; cumulative variance explained = 50.084%). CFA confirmed that the three-factor model fit well (*χ*^2^*/df* = 2.798, RMSEA = 0.065, CFI = 0.946, TLI = 0.937, GFI = 0.906, NFI = 0.919), and its fit was superior to that of the one-factor and two-factor models. The higher-order model was mathematically equivalent to the first-order three-factor model, and the bifactor model did not yield clearly interpretable specific factors. The first-order three-factor model was therefore retained as the most parsimonious and interpretable solution. Full measurement invariance across gender was supported (configural, metric, scalar, and strict invariance), whereas strict invariance across grade level was partially supported. MGT analysis yielded an overall scale generalizability coefficient of 0.948 and a reliability coefficient of 0.947, indicating high measurement precision across the three dimensions. The scale demonstrated excellent internal consistency (Cronbach’s *α* = 0.935) and test–retest reliability (*r* = 0.894). Validity analyses revealed acceptable convergent validity (AVE = 0.510–0.610), content validity (I-CVI ≥ 0.78; S-CVI/Ave = 0.91), and criterion-related validity (*r* = 0.771, *p* < 0.001).

**Conclusion:**

The HSSS demonstrates acceptable psychometric properties, including evidence of structural validity, high measurement precision, and measurement invariance across gender and grade level. The core three-factor structure was broadly replicated in an independent sample, although the fit was somewhat less optimal than in the primary sample. As a standardized and reliable psychometric instrument, it is suitable for quantitative shyness assessment and related empirical research among Chinese high school populations.

## 1 Introduction

### 1.1 Conceptualization of shyness

Shyness is a common yet complex psychological phenomenon in interpersonal interactions. It is generally defined as a state of discomfort and inhibition in social situations, characterized by excessive self-focus and fear of negative evaluation (Cheek and Buss, 1981; Crozier, 1979). Conceptually, shyness is a multifaceted psychobehavioral trait encompassing cognitive, affective, and behavioral components (Zimbardo, 1986, p. 3). From a historical perspective, early physiological accounts defined shyness as a state of high physical and mental inhibition in response to unfamiliar stimuli, characterized by blushing, rapid heartbeat, and physical incoordination (Lewinsky, 1941). Subsequent theoretical work shifted the focus to affective and behavioral components, describing shyness as a form of social anxiety accompanied by withdrawal and self-consciousness (Leary, 1986).

While early research often grouped shyness with introversion and social anxiety under the broader umbrella of social withdrawal, contemporary work has increasingly emphasized their distinct phenomenological and motivational signatures. Introversion primarily reflects a preference for solitary activities and a lower need for social stimulation; introverted individuals may enjoy solitude without experiencing fear or distress in social situations. In contrast, shyness involves an approach-avoidance conflict—a desire to interact coupled with a fear of doing so (Poole and Schmidt, 2020). This distinction is theoretically important: a shy individual may deeply wish to engage with others but is inhibited by negative self-evaluations and anticipated rejection. Similarly, while social anxiety shares features of fear and avoidance in social situations, shyness is often considered a broader temperamental trait that can include conflicted shyness (Mundelsee and Jurkowski, 2024). Social anxiety typically centers on intense fear of negative evaluation in performance or social settings, whereas shyness encompasses a wider range of affective, cognitive, and behavioral responses, including wariness in novel situations and self-consciousness in everyday interactions.

The present study conceptualizes shyness as a multidimensional trait in which individuals form negative self-cognitive evaluations, experience negative emotions such as tension and anxiety, and exhibit behavioral inhibition or social avoidance in specific social situations. This conceptualization is broadly consistent with the conflicted shyness tradition. However, it should be noted that the HSSS primarily captures the shyness dimension and does not directly assess the approach or sociability component. As a result, the scale does not distinguish between conflicted and avoidant shyness, as defined in the 2 × 2 model of shyness and sociability (Asendorpf, 1990).

### 1.2 Cultural and developmental contexts of shyness in Chinese adolescents

The expression and evaluation of shyness are deeply embedded in cultural contexts, and China offers a particularly illustrative case. Cultural self-construal theory provides a foundational explanation: Western cultures advocate independent self-construal emphasizing individual uniqueness, whereas Eastern cultures prioritize interdependent self-construal stressing connected relationships (Markus and Kitayama, 1991; Xu and Lin, 2026). In traditional Chinese society, influenced by Confucian values such as the “Doctrine of the Mean” and interpersonal harmony, shy and reserved behaviors were historically viewed as signs of modesty, self-restraint, and social maturity (Chen, 2013; Yu and Sun, 2024). Traditional family education reinforced these orientations by encouraging obedience and discouraging prominent self-expression (Liu et al., 2025; Yan, 2025), while widely shared beliefs—such as “going too far is as bad as not going far enough” and “outstanding people are vulnerable to criticism”—permeated everyday interactions and shaped a cultural logic in which shyness was not merely tolerated but often positively evaluated (Xu and Lin, 2026).

However, this cultural landscape has gradually evolved. With rapid societal modernization and global cultural integration, public perceptions and evaluations of shyness have progressively shifted, and the tendency to reduce shyness has increasingly become a mainstream social value orientation. This shift is driven by the growing demands of modern social and professional life. Modern professional systems place clear requirements on open communication, cooperative interaction, and active social participation (Umemura and Kao, 2026; Zhang et al., 2024). In contrast, the core features of shyness—social withdrawal, interaction avoidance, and poor cooperation—are increasingly divergent from modern expectations for active social engagement, making the reduction of shyness an increasingly recognized social expectation (Millett et al., 2026). Meanwhile, shyness can cause physical and mental discomfort such as rapid heartbeat, confused thinking, and facial flushing (Wang and Zhao, 2026), weakening social functions and restricting social development. Consequently, shyness has gradually been regarded as a negative psychological trait hindering interpersonal adaptation, a perception that extends to how shy individuals are viewed by others (Lacroix et al., 2025). Cross-cultural studies have reported that the overall shyness level of Asian groups tends to be higher than that of Western groups, and Chinese adolescents with high shyness appear to be more prone to developmental difficulties in campus adaptation and social interaction (Dong et al., 2024; Zhu et al., 2026). It is important to note, however, that many of these findings were derived from unidimensional measures, which may not fully capture the multidimensional nature of shyness. These cross-cultural comparisons should therefore be interpreted with appropriate caution, and they underscore the need for multidimensional instruments such as the HSSS.

The tension between traditional and modern expectations is particularly salient for adolescents growing up in this culturally transitional period. This tension is especially evident during the senior high school years (ages 14–19), a developmental period marked by critical transitions in identity formation, emotional regulation, and social adaptation (Erikson, 1968). Shyness during this period is not transient: it shows moderate to high rank-order stability across adolescence (Lawson et al., 2023), and evidence from earlier stages suggests that shyness levels tend to increase from infancy through early adolescence (Karevold et al., 2012). When such stability persists, excessive shyness may contribute to social difficulties, emotional anxiety, and other psychological problems, with potential long-term consequences for both current growth and later development (Chiu et al., 2025). Therefore, a culturally and developmentally sensitive measurement tool is needed to accurately assess the multidimensional nature of shyness in this population—one that captures not only behavioral withdrawal but also the cognitive and affective processes as they manifest within the Chinese high school context.

### 1.3 Limitations of existing shyness measures for Chinese high school students

Despite the availability of various shyness scales, existing measurement tools show notable limitations for assessing contemporary Chinese high school students. These limitations can be categorized as conceptual, developmental, and cultural in nature, each of which constrains the validity of shyness assessment in this population.

In terms of conceptual scope, widely used Western scales, such as the Cheek and Buss Shyness Scale (Cheek and Buss, 1981), rely on a unidimensional framework. While useful for estimating global shyness levels, they may not fully capture the heterogeneous internal mechanisms of shyness—specifically, the distinct cognitive, emotional, and behavioral manifestations that characterize this construct (Crozier, 2005). This limits their utility for fine-grained mechanistic research and for understanding how shyness operates across different social contexts. In studies of Chinese adolescents, commonly used measures include the Chinese version of the Cheek and Buss Shyness Scale (Cheek and Buss, 1981; Xiang et al., 2018) and peer-assessment measures such as the Revised Class Play (Masten et al., 1985). These instruments have provided useful evidence on the general level of shyness in Chinese samples, but they remain limited in their ability to capture the multidimensional internal mechanisms of shyness.

In terms of developmental fit, existing indigenous Chinese scales are not well suited to high school students. Scales developed for children (Wu, 2012) and junior high school students (Chen, 2013), both of which include child or adolescent shyness scale development, may not account for the unique social pressures of senior high school, while scales for college students (Wang, 2007) assume a level of social maturity and independence not yet developed in high schoolers. Currently, there appears to be a lack of a psychometrically validated, multidimensional shyness scale specifically designed for Chinese senior high school students.

In terms of cultural adaptation, the limitations of existing measures raise an important methodological concern: if previous instruments are indeed conceptually or psychometrically inadequate for this population, findings derived from them—including reported cross-cultural differences in shyness—may partly reflect measurement artifacts rather than genuine psychological differences. This further underscores the need for a culturally and developmentally appropriate measurement tool.

Together, these conceptual, developmental, and cultural limitations reveal a notable gap: existing instruments are conceptually narrow, developmentally mismatched, and lack cultural adaptation for Chinese high school students. Addressing this gap requires an instrument that is multidimensional in structure, developmentally tailored to adolescence, and culturally adapted to the Chinese high school context. The present study was developed in response to this need.

## 2 Methods

### 2.1 Study design and participants

This study followed established procedures for scale development. Participants were recruited across different phases to conduct successive investigations, including an open-ended questionnaire survey, item screening, exploratory structural analysis, confirmatory structural verification, reliability and validity examination, and test–retest stability assessment. All participants were senior high school students enrolled in regular high schools in Chongqing, China. Cluster sampling combined with convenience sampling was adopted for participant selection. To ensure independence across phases, separate samples were recruited for each stage, with no overlap between samples. Detailed demographic information for each sample is presented below.

#### 2.1.1 Sample 1: open-ended questionnaire survey

Grade 10 to Grade 12 students were recruited from one regular high school in Chongqing via cluster sampling. A total of 150 questionnaires were distributed, and 138 valid responses were retrieved, yielding an effective response rate of 92%. The sample consisted of 48 Grade 10 students, 50 Grade 11 students, and 40 Grade 12 students, with 63 males and 75 females. This sample was used to collect behavioral manifestations, core characteristics, situational features, and subjective experiences of shyness among senior high school students, providing indigenous empirical evidence for the compilation of initial scale items.

#### 2.1.2 Sample 2: exploratory factor analysis

Convenience sampling was used to recruit students from two regular high schools in Chongqing for the administration of the preliminary scale. In total, 500 questionnaires were distributed and 492 were returned. After excluding invalid questionnaires with regular answering patterns and excessive missing values, 487 valid questionnaires were retained, with an effective response rate of 97.40%. Among valid participants, there were 223 males and 264 females; 196 Grade 10 students, 173 Grade 11 students, and 118 Grade 12 students. This sample was used for scale item screening, item analysis, and exploratory factor analysis to preliminarily explore the dimensional structure of the scale.

#### 2.1.3 Sample 3: confirmatory factor analysis and reliability and validity examination

The formal scale was administered to students from two regular high schools in Chongqing using convenience sampling. A total of 450 questionnaires were issued and 435 were collected, and 427 valid questionnaires were finally obtained after screening, with an effective response rate of 94.89%. The sample included 172 males and 255 females, namely 190 Grade 10 students, 132 Grade 11 students, and 105 Grade 12 students. This sample was mainly used to verify the structural stability of the scale, conduct confirmatory factor analysis, multivariate generalizability analysis, measurement invariance tests, and overall psychometric assessment.

#### 2.1.4 Sample 4: test–retest reliability

To examine the temporal stability of the scale, one class from each grade in a regular high school in Chongqing was selected as retest participants via convenience sampling. Initially, 180 students participated in the first measurement. After excluding dropouts and invalid cases, 176 valid participants remained, including 77 males and 99 females. The second round of measurement was conducted after a four-week interval, and this sample was used to calculate the test–retest reliability coefficient.

#### 2.1.5 Sample 5: criterion-related validity

A total of 200 senior high school students from one regular high school in Chongqing were recruited as participants for criterion-related validity testing through convenience sampling, covering students from all grades. The valid sample contained 102 males and 98 females, with 86 Grade 10 students, 62 Grade 11 students, and 52 Grade 12 students. This sample was used to implement criterion-related validity analysis and verify the practical effectiveness and predictive power of the developed scale. The Cheek and Buss Shyness Scale was administered as the criterion measure. In addition to criterion-related validity, Sample 5 also provided item-level HSSS data for a supplementary cross-validation of the final 18-item model.

All participating schools were located in district and county-level high schools in Chongqing, China. Students in these schools came from both urban and rural household registration backgrounds. These schools were selected based on their accessibility and willingness to participate, and they represent typical district and county-level high schools in the region in terms of student demographics and academic performance. The sample thus reflects the characteristics of high school students in Chongqing, but may not fully represent students from other provinces or regions of China.

### 2.2 Scale development

#### 2.2.1 Establishment of scale dimensions

Shyness is a multifaceted psychological trait defined by intertwined cognitive, emotional, behavioral, and situational features, rather than a unitary behavioral phenomenon, which substantiates its inherent multidimensional structure. Given such structural complexity, conventional unidimensional measurement approaches may not fully capture the core connotation and intricate underlying mechanisms of shyness. As discussed in the Introduction, existing shyness measures have notable limitations for Chinese high school students, particularly their unidimensional structure and lack of developmental and cultural adaptation. To address these methodological deficiencies, existing scholarship has gradually moved beyond unidimensional paradigms and empirically validated the multidimensional nature of shyness, forming relatively systematic dimensional classification frameworks. Early studies concentrated on discrete core components of shyness: Alm and Lindberg (1999) highlighted affective and cognitive characteristics, regarding social embarrassment and fear of negative evaluation as central shy features, whereas Crozier (2005) established an integrated measurement system incorporating cognitive, emotional, and behavioral indicators. On this basis, subsequent research further improved dimensional construction by incorporating situational triggers and developmental mechanisms. A comprehensive multidimensional structure grounded in the full social interaction process was developed by Henderson et al. (2010). The social adaptation model conceptualizes shyness as a dynamic process unfolding across three temporal stages: social approach, real-time interaction, and post-interaction adjustment. The HSSS dimensions, by contrast, are organized along the cognitive, affective, and behavioral components of shyness. These two frameworks are not in conflict, but complementary. Each stage of the social interaction process may involve all three components to varying degrees. For instance, negative self-consciousness (the cognitive component) may be activated before, during, and after social interaction; negative emotional experience (the affective component) may arise throughout the entire process; and withdrawal from avoidant speech and behavior (the behavioral component) may occur at any stage. The HSSS thus integrates the component-based structure of shyness with the temporal dynamics emphasized by the social adaptation model, offering a more comprehensive framework for understanding shyness among Chinese high school students. Cumulative empirical evidence further supports that shyness arises from the interplay of multiple psychological and behavioral factors. Therefore, unidimensional models may be insufficient to account for its diverse manifestations and complex internal mechanisms, supporting the necessity of multidimensional scale development (Amendola and Cerutti, 2023; Beckert et al., 2025).

Building on these multidimensional theoretical foundations, the present study adopted Henderson et al.'s (2010) social adaptation model of shyness as the core theoretical framework and integrated the localized scale development strategy proposed by Wang (2007). In accordance with the physical and psychological developmental traits, typical campus social contexts, and indigenous cultural characteristics of Chinese senior high school students, rigorous dimension screening and refinement were conducted. This localized optimization addresses the limited cross-cultural applicability of Western shyness scales and the insufficient targeting of general adolescent scales for senior high school populations. Ultimately, three core dimensions were determined: Withdrawal from avoidant speech and behavior, Negative self-consciousness, and Negative emotional experience. This tripartite structure was preferred over alternative dimensional divisions because a unidimensional model would fail to differentiate the cognitive, affective, and behavioral components of shyness, whereas a two-dimensional model would omit either the affective or the behavioral dimension. Compared with existing instruments, the present scale integrates internal psychological mechanisms and external behavioral performances, covers the complete social interaction process, and fits the indigenous developmental characteristics of Chinese senior high school students, enabling more accurate evaluation of the internal structure and developmental traits of shyness in this population (see Figure 1).

**Figure 1:**
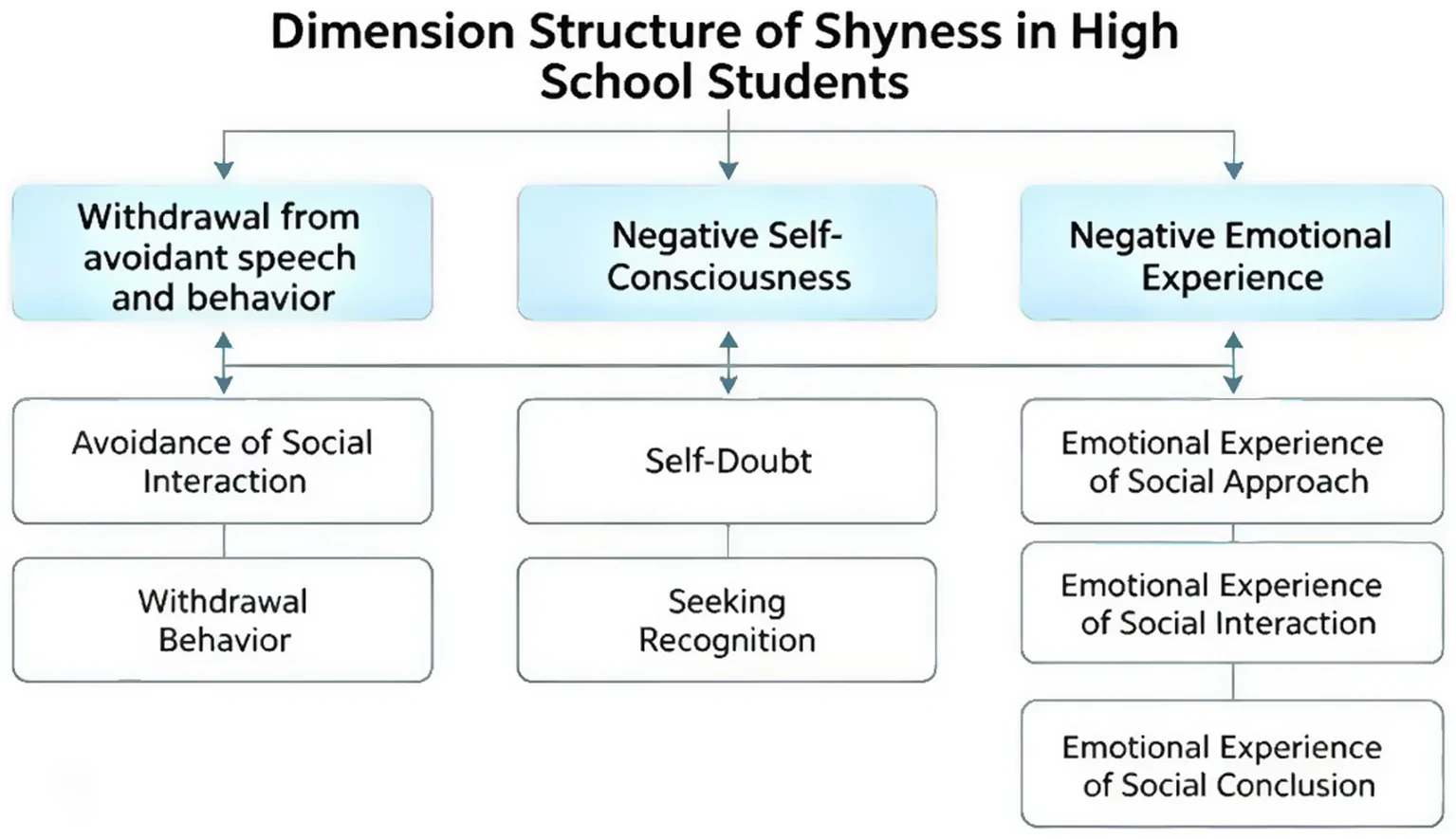
Dimension structure of the High School Student Shyness Scale (HSSS). This figure illustrates the three-factor structure of the HSSS: Withdrawal from avoidant speech and behavior, Negative self-consciousness, and Negative emotional experience. Each dimension is further specified by its typical manifestations: avoidance of social interaction and withdrawal behavior (for the first dimension), self-doubt and seeking recognition (for the second dimension), and emotional experiences across the social approach, real-time interaction, and post-interaction stages (for the third dimension).

#### 2.2.2 Preparation of open-ended questionnaires

To further refine the dimensional connotation of shyness and consolidate the theoretical foundation for scale construction, this study conducted an open-ended questionnaire survey among Grade 10 to Grade 12 students in the preliminary stage of scale development. This qualitative investigation aimed to systematically collect localized empirical data regarding Chinese senior high school students’ typical shy behavioral manifestations, high-frequency social contexts, and core triggering factors of shyness. First, through a comprehensive review of domestic and international studies on shyness, the current research clarified the core connotation and preliminary structural framework of shyness. Combined with professional guidance from psychological experts, a targeted open-ended questionnaire tailored for senior high school students was subsequently developed. The questionnaire consisted of three core items: (1) Do you have shy classmates? In what kinds of situations do they usually exhibit shy characteristics? (2) What behavioral or psychological responses can be regarded as typical manifestations of shyness? (3) In your view, what are the primary factors that trigger shy psychology? By integrating firsthand empirical data obtained from the open-ended survey and existing theoretical achievements, this study preliminarily established a psychological structural model of shyness for senior high school students. The findings provide empirical support and a realistic basis for subsequent scale item compilation, dimensional verification, and structural optimization.

#### 2.2.3 Development of the preliminary scale

Based on the theoretical framework established in the preliminary stage and the dimensional structure derived from open-ended investigations, item compilation and screening for the preliminary shyness scale were systematically conducted. Indigenous items tailored to the psychological developmental characteristics of senior high school students were developed in accordance with the typical shy behavioral features and campus social scenarios summarized from open-ended questionnaire data. Meanwhile, previously validated items from existing domestic and international shyness scales were adopted and localized through revisions that fit the daily campus situations and group traits of Chinese senior high school students, ensuring that all compiled items were grounded in solid theoretical evidence and possessed adequate content validity. Multiple rounds of item evaluation and optimization were performed jointly by in-service senior high school teachers and professional psychology experts. Items with ambiguous expressions, repetitive semantics, and unrealistic campus situational descriptions were eliminated, while items with awkward phrasing and poor contextual adaptability were revised accordingly. After systematic item screening and wording modification, a total of 42 items were retained for the preliminary scale. The scale adopts a five-point Likert scoring method, with response options ranging from “strongly disagree,” “disagree,” “uncertain,” “agree” to “strongly agree,” corresponding to scores of 1 to 5. Participants responded based on their real-life experiences, enabling quantitative assessment of individual shyness levels. The full preliminary scale is provided in Appendix A.

To ensure transparency in item development, the preliminary 42 items were generated from three sources: (a) 30 indigenous items derived from open-ended questionnaire responses collected from 138 high school students; (b) 6 items adapted from existing domestic and international shyness scales (Cheek and Buss, 1981; Wang, 2007; Chen, 2013); and (c) 6 items generated and refined by an expert panel of psychology researchers and in-service high school teachers based on theoretical considerations and student feedback. Importantly, although several items were adapted from existing shyness scales, these scales were not adopted in their entirety. The Cheek and Buss (1981) Shyness Scale is unidimensional and does not differentiate between the cognitive, affective, and behavioral components of shyness; the Wang (2007) scale was developed for college students and assumes a level of social maturity not yet developed in high schoolers; and Chen (2013) scale was designed for junior high school students and focuses on adolescent social contexts (e.g., peer interactions and classroom participation) that do not capture the complex social situations of high school students (e.g., public speaking, opposite-sex interactions, peer evaluation). Therefore, only those items that reflected core and developmentally appropriate manifestations of shyness were adapted, and all adapted items were further localized and refined through expert review to fit the Chinese high school context. Following expert review for clarity, relevance, and contextual appropriateness, all items were subjected to empirical screening. After exploratory and confirmatory factor analyses, the final 18-item HSSS retained 14 indigenous items (77.8%), 1 item adapted from Cheek and Buss (1981) (5.6%), 1 item adapted from Wang (2007) (5.6%), 1 item adapted from Chen (2013) (5.6%), and 1 expert-revised item (5.6%). The final HSSS is thus a multidimensional, developmentally tailored, and culturally grounded instrument rather than a simple translation or adaptation of any single existing scale. A complete record of item origins, modifications, and screening outcomes is provided in Appendix B.

#### 2.2.4 Determination of formal scales

Item analysis and exploratory factor analysis were carried out on the data of the preliminary scale and confirmatory factor analysis, generalizability analysis, and reliability and validity examination were conducted, as detailed in Appendix B.

### 2.3 Data analysis strategy

To address the methodological limitations of previous shyness measures and to establish the psychometric properties of the HSSS, a series of statistical analyses were conducted using SPSS 26.0, Amos 26.0, and mGENOVA.

Item analysis. Item discrimination was evaluated using the critical ratio method (comparing the top 27% and bottom 27% of total scores via independent-samples *t*-tests) and item-total correlations. Items with non-significant *t*-values (*p* > 0.05) or item-total correlations below 0.40 were removed.

Exploratory factor analysis (EFA). To identify the latent structure of shyness, Principal Axis Factoring (PAF) was employed as the extraction method. Unlike principal component analysis (PCA), PAF is a common factor model that estimates latent factors by analyzing shared variance among items, making it particularly suitable for scale development (Fabrigar et al., 1999). Given the theoretical expectation that the dimensions of shyness would be interrelated, Promax oblique rotation was applied to allow for correlations among factors. For factor retention, multiple criteria were employed: (a) eigenvalues greater than 1.0, (b) visual inspection of the scree plot, and (c) Parallel Analysis (PA), which is increasingly recommended as a robust method for determining the number of factors to retain (Glorfeld, 1995; Horn, 1965). Items with communalities below 0.40, factor loadings lower than 0.50, or cross-loadings without clear theoretical interpretation were systematically removed.

Confirmatory factor analysis (CFA). To verify the structural stability of the three-factor model and to examine whether a general shyness factor dominates the structure, seven competing models were tested: (a) a one-factor model, (b) three two-factor models, (c) a first-order three-factor model, (d) a higher-order factor model, and (e) a bifactor model. All models were estimated using maximum likelihood estimation in Amos 26.0. Model fit was evaluated using multiple indices: *χ*^2^/df < 3, RMSEA<0.08, RMR < 0.08, and GFI, TLI, CFI, NFI, IFI, RFI > 0.90. Items with standardized factor loadings below 0.50 were removed based on both statistical and theoretical considerations. The best-fitting and most parsimonious model was retained. In addition, a supplementary cross-validation of the final 18-item model was conducted using Sample 5 (*N* = 200), which contained item-level HSSS data. Model fit was evaluated using the same criteria as above.

Reliability and validity. Internal consistency was assessed using Cronbach’s *α* and composite reliability (CR). For the Negative emotional experience (F3) dimension, the retained 9 items demonstrated standardized factor loadings ranging from 0.68 to 0.82, and Cronbach’s *α* was 0.923, indicating strong internal consistency. Test–retest reliability was evaluated using Pearson correlations over a four-week interval. Convergent validity was assessed using average variance extracted (AVE), and discriminant validity was examined by comparing the square root of AVE with inter-factor correlations. Criterion-related validity was evaluated by correlating the HSSS total score with the Cheek and Buss Shyness Scale. Multivariate generalizability theory (MGT) analysis was conducted using mGENOVA to evaluate measurement precision across facets.

Qualitative coding of open-ended responses. The open-ended questionnaire responses were independently coded by two trained researchers based on the pre-established three-dimensional theoretical model (Withdrawal from avoidant speech and behavior, Negative self-consciousness, and Negative emotional experience). To assess inter-coder reliability, a randomly selected subset of 40 responses was double-coded by both researchers. Cohen’s Kappa was calculated using SPSS, which yielded a Kappa value of 0.71, indicating substantial agreement (Landis and Koch, 1977). Disagreements were resolved through discussion until consensus was reached.

### 2.4 Measurement process and data analysis

Prior to formal data collection, all questionnaire instructions, response guidelines, and administration protocols were fully standardized to ensure consistent testing criteria across all participants. Data collection was implemented in group units by class. After obtaining formal informed consent from participating schools, teachers, students, and their parents, trained testers uniformly delivered standardized instructions and precautions, and guided participants to complete the questionnaires independently and truthfully. All questionnaires were completed and collected on-site, which effectively minimized missing data and erroneous responses and ensured the authenticity and completeness of the original dataset. Following systematic data sorting and cleaning, the data were analyzed using SPSS 26.0, Amos 26.0, and mGENOVA.

## 3 Results

### 3.1 Open-ended questionnaire analysis

To empirically validate the scientificity and contextual adaptability of the pre-established three-dimensional theoretical framework of shyness and further consolidate the theoretical foundation for scale development, this study conducted systematic collation, qualitative coding, and categorical analysis on open-ended questionnaire data from senior high school students. The results indicated that all shy behavioral manifestations, negative cognitive characteristics, and adverse emotional experiences reported by participants could be categorized into the three predefined dimensions of shyness: Withdrawal from avoidant speech and behavior, Negative self-consciousness, and Negative emotional experience. Throughout the qualitative analysis, no unclassifiable responses or newly emergent dimensions were identified. The core definitions, typical manifestations, and corresponding frequencies of each dimension are summarized in Table 1.

**Table 1:** List of results of open-ended questionnaires (partial).

Theoretical dimension	Dimension definition	Typical manifestation items and statistical frequencies
Withdrawal from avoidant speech and behavior	In diverse campus interpersonal scenarios, individuals tend to exhibit prominent external behavioral inhibition, including active social avoidance, verbal restraint, and interactive withdrawal. Such behavioral patterns primarily stem from worries about poor social performance and fear of negative interpersonal evaluation, which constitute the typical behavioral externalization of shyness among senior high school students.	Avoiding campus social occasions (56), withdrawing from uncomfortable interpersonal situations (47), restrained and introverted verbal expression (45), avoiding eye contact during social interaction (7), tending to leave ongoing social interactions (9), escaping from oppressive interpersonal contexts (18), evading face-to-face interpersonal communication (12), rigid and reserved social state (25), stiff and constrained body postures in social situations (10), nervous and restrained small movements caused by social tension (34)
Negative self-consciousness	During interpersonal communication and social interaction, individuals with shy traits are excessively sensitive to external evaluations and prone to overestimating their own social deficiencies, thereby forming stable negative cognitive biases and persistent self-denial tendencies. This negative cognitive feature acts as the core internal cognitive inducement that triggers shy behavioral patterns in senior high school students.	Doubting personal social competence (45), negatively evaluating personal social performance (67), experiencing inferiority during social interaction (14), perceiving one’s social performance as inferior to others (34), denying personal interpersonal value (69), worrying about negative social impressions after interaction (80)
Negative emotional experience	Throughout the whole-stage social process covering social approach, real-time interaction, and post-interaction adjustment, individuals continuously experience negative emotions such as tension, anxiety, panic and unease due to fear of potential social risks and excessive preoccupation with others’ attention. This stable negative emotional response serves as the distinctive internal emotional manifestation of senior high school students’ shyness traits.	Fearing denial and criticism in social situations (64), worrying about interpersonal isolation caused by verbal errors (4), dreading being the focus of public attention (34), feeling anxious about inappropriate performance before social interaction (12), experiencing tension and panic during social interaction (18)

Frequency counts represent the number of participants (out of 138) who endorsed each manifestation item. Only partial results are shown; complete results are available from the authors.

### 3.2 Item analysis

To rigorously screen qualified items with robust discriminative power and favorable psychometric quality for scale optimization, this study conducted systematic item analysis on the initial 42 scale items via the critical ratio method and item-total correlation method, so as to complete standardized preliminary item filtering.

Critical ratio method. Participants’ total shyness scores were sorted in hierarchical order, with respondents falling in the top 27% and bottom 27% of the score distribution categorized into the high-score group and low-score group, respectively. Independent-samples *t*-tests were conducted to compare intergroup differences in individual item scores. Analytical results demonstrated that the majority of items reached a significant difference level of *p* < 0.001. Nevertheless, Item 37 showed non-significant differences between the two groups (*p* = 0.17 > 0.05), and Item 39 presented a low *t*-value and poor item discrimination efficiency. In line with standard psychometric guidelines for scale development, Item 37 and Item 39 were eliminated, and 40 items were reserved for the subsequent item-total correlation analysis (Wu, 2010).

Item-total correlation method. On the basis of the reserved 40 items, Pearson product–moment correlation analysis was further implemented to assess the correlation degree between each single item and the overall scale score. The results revealed that six items (Item 4, Item 11, Item 38, Item 40, Item 41, and Item 42) possessed correlation coefficients lower than the widely recognized critical value of 0.40. All six unqualified items were excluded, and a total of 34 valid items were ultimately retained for follow-up dimensional verification and structural optimization of the scale.

### 3.3 Exploratory factor analysis

The Kaiser–Meyer–Olkin (KMO) measure of sampling adequacy was 0.947, and Bartlett’s test of sphericity was significant (*χ*^2^ = 8,910.683, *df* = 561, *p* < 0.001), confirming the suitability of the data for factor analysis. The 34 items retained after item analysis were then subjected to Principal Axis Factoring (PAF) with Promax oblique rotation. Parallel analysis strongly supported a three-factor solution: the first three eigenvalues from the actual data (13.080, 2.366, and 1.583) exceeded the 95th percentile of eigenvalues generated from random data (1.611, 1.506, and 1.455, respectively), whereas the fourth eigenvalue (1.285) fell below its corresponding random threshold (1.406). The scree plot also clearly indicated a three-factor structure, with a marked elbow after the third factor (see Figure 2). The three extracted factors cumulatively explained 50.084% of the total variance. The Promax rotation yielded a factor correlation matrix showing moderate-to-high correlations among the three factors (r = 0.586 to 0.744), confirming the appropriateness of the oblique rotation. Based on communalities below 0.40, factor loadings below 0.50, or cross-loadings above 0.40, items were iteratively removed (Hair et al., 2009; Worthington and Whittaker, 2006). This process resulted in 21 items being retained in the initial three-factor structure: Factor 1 (Withdrawal from avoidant speech and behavior; 6 items), Factor 2 (Negative self-consciousness; 6 items), and Factor 3 (Negative emotional experience; 9 items). These 21 items were subsequently entered into the confirmatory factor analysis (see Section 3.4.1). The pattern matrix is presented in Table 2. The full results of the parallel analysis are presented in Supplementary Figure S1 and Supplementary Table S1.

**Figure 2:**
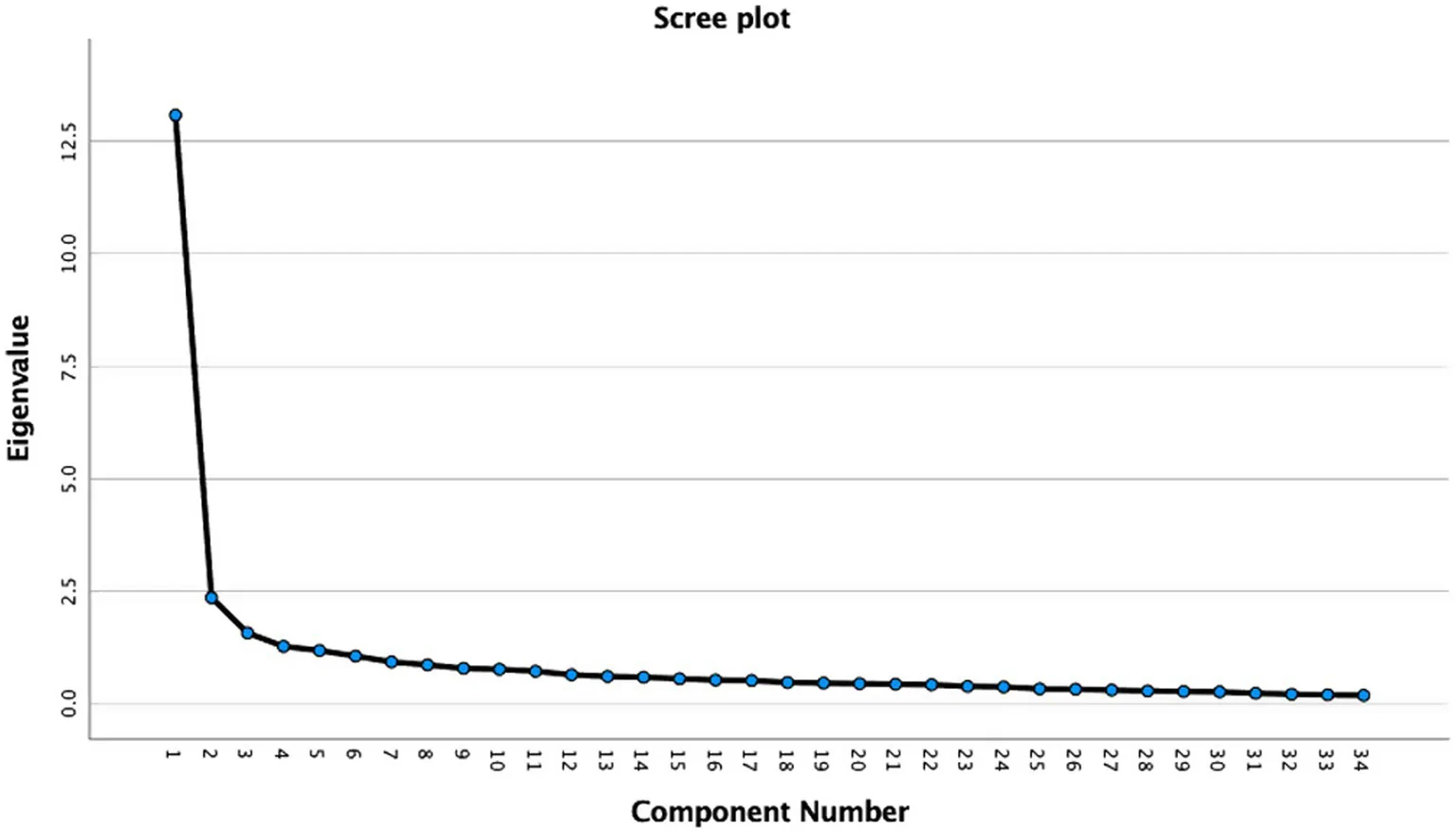
Scree plot of the High School Student Shyness Scale (HSSS). The scree plot shows the eigenvalues of extracted factors, supporting the three-factor structure.

**Table 2:** Exploratory factor analysis form.

Item	Factor 1	Factor 2	Factor 3
S3	0.770		
S6	0.615		
S8	0.753		
S9	0.692		
S10	0.600		
S27	0.711		
S15		0.577	
S18		0.600	
S19		0.689	
S20		0.872	
S22		0.689	
S23		0.674	
S26			0.632
S28			0.548
S29			0.831
S30			0.697
S31			0.654
S32			0.824
S33			0.901
S34			0.627
S35			0.784
Eigenvalue	1.583	2.366	13.080
Variance contribution (%)	4.655	6.960	38.470
Total variance explained (%)	50.084		

Extraction method: Principal Axis Factoring. Rotation method: Promax with Kaiser Normalization. The eigenvalues and variance percentages reported in this table are based on the initial 34-item correlation matrix. Factor 1 = Withdrawal from avoidant speech and behavior; Factor 2 = Negative self-consciousness; Factor 3 = Negative emotional experience.

### 3.4 Confirmatory factor analysis

#### 3.4.1 Initial and final models

The initial baseline model, which included all 21 items retained from EFA, showed suboptimal fit (see Table 3): χ^2^/df = 3.689, RMSEA = 0.079, CFI = 0.905, TLI = 0.893, GFI = 0.861, NFI = 0.875, RFI = 0.859. Inspection of the modification indices and standardized factor loadings revealed that three items (Item 15, Item 23, and Item 27) exhibited factor loadings below the 0.50 threshold (0.336, 0.271, and 0.436, respectively). These three items were removed based on both statistical and theoretical considerations. Theoretically, all three items were characterized by ambiguous or redundant wording: Item 15 was semantically redundant with other items in the Negative self-consciousness dimension; Item 23 overlapped substantially with Item 20; and Item 27, although it loaded on Withdrawal from avoidant speech and behavior in the EFA, was theoretically inconsistent with this behavioral dimension and exhibited a low standardized factor loading (0.436) in the CFA. After removing these items, the modified 18-item model was re-fitted and showed good fit: χ^2^/df = 2.798, RMSEA = 0.065, CFI = 0.946, TLI = 0.937, *GFI* = 0.906, *NFI* = 0.919, *RFI* = 0.906. After deletion, the Negative emotional experience dimension (F3) retained 9 items with factor loadings ranging from 0.68 to 0.82, demonstrating acceptable internal consistency (Cronbach’s *α* = 0.923). These results support the structural stability of the three-factor model and provide evidence for the construct validity of the HSSS. The final model is presented in Figure 3.

**Table 3:** Indicators of model fit for shyness scale model for high school students.

Model	CMIN	*df*	*χ*^2^/*df*	*p*	RMR	TLI	CFI	GFI	NFI	IFI	RFI	RMSEA
Initial (21 items)	686.150	186	3.689	0.000	0.103	0.893	0.905	0.861	0.875	0.906	0.859	0.079
Final (18 items)	369.357	132	2.798	0.000	0.061	0.937	0.946	0.906	0.919	0.946	0.906	0.065

Initial model = 21-item three-factor model before item deletion; Final model = 18-item three-factor model after removing Items 15, 23, and 27. All indices were estimated using maximum likelihood in Amos 26.0. The initial model demonstrated suboptimal fit across multiple indices, which justified the removal of the three items and the subsequent retention of the final 18-item model.

**Figure 3:**
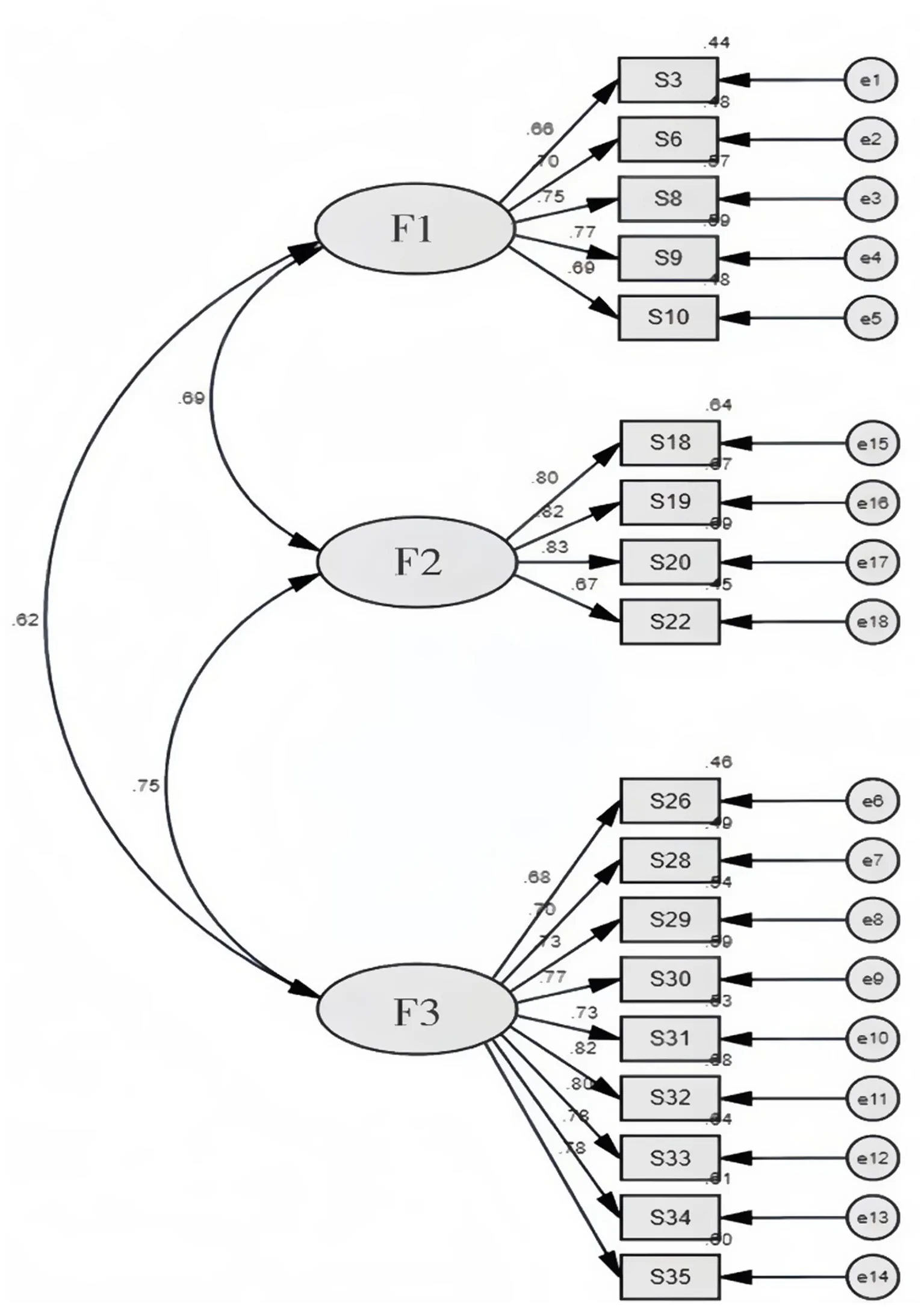
Confirmatory factor analysis of the three-factor shyness model. Standardized path coefficients for the three-factor model of the High School Student Shyness Scale (HSSS). All factor loadings are significant at *p* < 0.001.

#### 3.4.2 Comparison of alternative structural models

To examine whether a general shyness factor dominates the structure, seven competing models were tested (see Table 4): (a) a one-factor model, (b) three two-factor models, (c) a first-order three-factor model, (d) a higher-order factor model, and (e) a bifactor model. The one-factor model showed poor fit (*χ*^2^/*df* = 7.541, CFI = 0.799, RMSEA = 0.124), and all three two-factor models also showed inadequate fit (CFI = 0.852–0.891, RMSEA = 0.092–0.107). The first-order three-factor model showed good fit (*χ*^2^/*df* = 2.798, CFI = 0.946, RMSEA = 0.065) and outperformed the one-factor model (Δ*χ*^2^ = 648.701, Δ*df* = 3, *p* < 0.01).

**Table 4:** Comparison of alternative structural models.

Number	Model	χ2	*df*	χ2/*df*	NFI	CFI	RMSEA	Model comparison	ΔX2	Δ*df*
1	Original model (First-order three-factor)	369.357	132	2.798	0.919	0.946	0.065	–	–	–
2	Two-factor model 1	673.436	134	5.026	0.852	0.877	0.097	2 vs. 1	304.079^**^	2
3	Two-factor model 2	614.655	134	4.587	0.865	0.891	0.092	3 vs. 1	245.298^**^	2
4	Two-factor model 3	789.832	134	5.894	0.827	0.852	0.107	4 vs. 1	420.475^**^	2
5	One-factor model	1018.06	135	7.541	0.776	0.799	0.124	5 vs. 1	648.701^**^	3
6	Higher-order model	369.357	132	2.798	0.919	0.946	0.065	6 vs. 1	0.00	0.00
7	Bifactor model	289.99	119	2.437	0.936	0.961	0.058 [0.050, 0.067]	7 vs. 1	79.37^**^	13

*N* = 427. ** denotes (*p* < 0.01), indicating statistically significant difference in chi-square values between the compared models. Original model = First-order three-factor model (Withdrawal from avoidant speech and behavior, Negative self-consciousness, Negative emotional experience). Two-factor model 1 = Withdrawal from avoidant speech and behavior + Negative self-consciousness, combined with Negative emotional experience. Two-factor model 2 = Withdrawal from avoidant speech and behavior + Negative emotional experience, combined with Negative self-consciousness. Two-factor model 3 = (Withdrawal from avoidant speech and behavior + Negative emotional experience) + Negative self-consciousness. One-factor model = all three dimensions combined.

To further examine the role of a general factor, a higher-order factor model and a bifactor model were tested. The higher-order model was mathematically equivalent to the first-order three-factor model (Δ*χ*^2^ = 0.00, Δ*df* = 0). This equivalence is a mathematical consequence of the just-identified structural model with three first-order factors, rather than evidence that a general factor lacks explanatory power. The substantial second-order loadings (F1 = 0.756, F2 = 0.915, F3 = 0.817) suggest that a common factor may account for a considerable proportion of the variance. The bifactor model showed good fit indices (*χ*^2^*/df* = 2.44, CFI = 0.961, RMSEA = 0.058), but the standardized factor loadings for the F3 dimension were negative (ranging from −0.508 to 0.009), and none of the unstandardized loadings for F3 were statistically significant (all *p* > 0.05). No inadmissible parameter estimates such as negative residual variances or standardized loadings exceeding 1 were observed. Nonetheless, the negative and non-significant loadings on the specific F3 factor suggest that F3 did not contribute meaningful variance beyond the general factor. Given that the first-order three-factor model (Original model) demonstrated good fit, the higher-order model was mathematically equivalent to it, and the bifactor model did not provide clearly interpretable specific factors, the first-order three-factor model was retained as the most theoretically parsimonious and interpretable solution.

To further evaluate the practical contribution of the general and specific factors, bifactor indices were computed (see Table 5). The results showed that the general factor accounted for the majority of common variance and total score reliability, whereas the specific factors provided limited reliable information beyond the total score. In particular, the specific factor loadings for F3 were negative and the factor variance was zero, indicating that F3 did not contribute meaningful variance beyond the general factor. It should be noted that the zero factor variance for F3 represents a boundary estimate, although no other inadmissible parameter estimates were observed. These results suggest that the subscale scores, especially F3, should be interpreted with caution, and that the total score may serve as the primary indicator of shyness. In addition, ω_total was 0.935, which is larger than ωH (0.929), as expected; the high ωH relative to Cronbach’s *α* (0.935) reflects the strong general factor in the bifactor solution. In sum, the bifactor model did not yield clearly interpretable specific factors, supporting the retention of the first-order three-factor model as the most parsimonious and interpretable solution.

**Table 5:** Bifactor indices for the general and specific factors.

Factor	ECV	ωH	ωHS	H
General factor	0.709	0.929	–	–
F1	–	–	0.457	0.635
F2	–	–	0.329	0.575
F3	–	–	n.a.	n.a.
ω_total	–	0.935	–	–

ECV = explained common variance; ωH = omega hierarchical; ω_total = omega total; ωHS = omega hierarchical subscale; H = construct replicability; n.a. = not applicable because the specific factor loadings for F3 were negative and the factor variance was zero.

#### 3.4.3 Cross-validation in an independent sample

To examine whether the final 18-item structure would replicate in an independent sample, a cross-validation was conducted using Sample 5 (*N* = 200), which was originally collected for criterion-related validity. Model fit was acceptable for *χ*^2^/*df* = 2.751, whereas CFI = 0.889, TLI = 0.871, and RMSEA = 0.094 fell slightly below conventional thresholds. Standardized loadings were generally adequate, although two items, S30 and S35, showed loadings of 0.320 and 0.403, both below the 0.50 threshold. Supplementary analyses excluding one or both of these items did not yield a substantial improvement in fit. These results suggest that the less optimal fit observed in Sample 5 may be attributable to differences in sample size and sampling procedure rather than to a fundamental misspecification of the three-factor structure, which was well supported in Sample 3 (*N* = 427), with CFI = 0.946, TLI = 0.937, and RMSEA = 0.065.

### 3.5 Formal questionnaire reliability analysis

#### 3.5.1 Reliability analysis based on multivariate generalization theory

##### 3.5.1.1 G-study with three-factor models

To evaluate the measurement precision of each dimension and to disentangle multiple sources of measurement error, multivariate generalizability theory (MGT) was applied to the formal shyness scale. A multivariate single-facet random cross design (*p* × *i*) was employed for the G-study, with participants (*p*) treated as the object of measurement and scale items (*i*) as the measurement facet. Participants and items were specified as fully crossed and random. Using mGENOVA, variance and covariance components attributable to participant effects, item effects, and participant × item interaction effects were separately estimated for the three-factor structure. Detailed variance and covariance components for each factor are presented in Table 6.

**Table 6:** Variance and covariance matrix of various effects on three factors.

Effect	Factor	F1	F2	F3
Participants (*p*)	F1	**0.751**		
	F2	0.540	**0.790**	
	F3	0.493	0.622	**0.835**
Items (*i*)	F1	**0.014**		
	F2		**0.024**	
	F3			**0.009**
Participants × items (*p* × *i*)	F1	**0.735**		
	F2		**0.520**	
	F3			**0.629**

Only the variance components (diagonal) and covariance components among the three factors for the participant effect are presented. Covariance components for the item and participant × item effects are not estimated, as they are not of theoretical interest in the present design. Therefore, the corresponding cells are left blank. Values in bold represent estimated diagonal variance components. F1 = Withdrawal from avoidant speech and behavior; F2 = Negative self-consciousness; F3 = Negative emotional experience.

The G-study results showed three main findings. First, the largest variance component was attributable to participants (ranging from 0.751 to 0.835 across the three dimensions), indicating that individual differences in shyness were adequately captured by the scale. Second, the item effect was negligible (ranging from 0.009 to 0.024), suggesting that no single item appeared to systematically bias the measurement—a finding that supports the content validity of the HSSS. Third, the participant × item interaction effect ranged from 0.520 to 0.735, reflecting some variability in how participants responded across items; this is common in psychological measurement and indicates that items are not perfectly interchangeable.

##### 3.5.1.2 D-study with three-factor models

Estimation accuracy of the full domain scores for each factor. The D-study results showed that the full domain variance components were 0.751 for F1, 0.790 for F2, and 0.835 for F3. Taking measurement error into account, the generalizability coefficients were 0.836 for F1, 0.859 for F2, and 0.923 for F3, with F3 (Negative emotional experience) demonstrating the highest measurement precision, followed by F2 (Negative self-consciousness) and F1 (Withdrawal from avoidant speech and behavior). These results indicate that all three dimensions achieved acceptable measurement precision, and the overall reliability of the HSSS was acceptable, supporting its use for both norm-referenced and criterion-referenced assessment. Detailed results are presented in Table 7. It should be noted that, in the single-facet *p* × *i* design, these generalizability coefficients are mathematically equivalent to Cronbach’s *α*. This equivalence notwithstanding, MGT offers distinctive advantages for evaluating measurement precision, particularly through its decomposition of measurement error and its capacity to model alternative measurement designs. These advantages are discussed further in the following section.

**Table 7:** A multivariate generalizability D-study of the three factors of shyness in high school students.

Index	F1	F2	F3
Full domain variance	0.751	0.790	0.835
Relative error variance	0.147	0.130	0.070
Absolute error variance	0.150	0.136	0.071
Mean error variance	0.005	0.008	0.003
Standard Error of the Full Range	0.866	0.889	0.914
Relative Error Standard Error	0.384	0.361	0.264
Absolute Error Standard Error	0.387	0.369	0.266
Standard Error of Mean	0.070	0.091	0.056
Generalizability coefficient	0.836	0.859	0.923
Reliability index	0.834	0.853	0.922
Relative signal-to-noise ratio	5.103	6.078	11.935
Absolute signal-to-noise ratio	5.010	5.807	11.767

Measurement accuracy of the total score of the whole domain. The weighting coefficients for synthesizing the full domain total score were determined according to the proportion of items in each dimension (F1 = 0.278, F2 = 0.222, F3 = 0.500), as shown in Table 8. By synthesizing the three factors, the variance component of the full domain total score was estimated to be 0.647, and the variance components of the relative and absolute errors were 0.035 and 0.036, respectively. The generalization coefficient of the full domain total score was 0.948, and the reliability index was 0.947, as shown in Table 9. These results indicate that the HSSS achieved acceptable measurement precision at the total score level, with relatively small error variance, supporting its use for both norm-referenced and criterion-referenced assessment.

**Table 8:** Weighting coefficients for the factors in the D-study to synthesize the total domain-wide score.

Factor	F1	F2	F3
Weighting coefficients (w)	0.278	0.222	0.500

**Table 9:** Estimation of variance components for total domain scores in the D-study.

Index	Estimated value
Variance component of the total score of the full domain	0.647
Variance Component of Relative Error for Full Domain Total Score	0.035
Variance Component of Absolute Error of Total Score in Full Domain	0.036
Variance component of the mean of the total score error in the whole domain	0.002
Standard deviation of total score	0.805
Standard deviation of the relative error of the global total score	0.188
Standard deviation of the absolute error of the domain total score	0.190
Standard deviation of the mean of the errors of the total score of the whole domain	0.049
Generalization coefficient of the global total score	0.948
Reliability index of the global total score	0.947
Relative signal-to-noise ratio of global total score	18.362
Absolute signal-to-noise ratio of total score	17.975

Contribution of each factor to the total variance. To examine the extent to which each dimension contributes to the total score variance of the HSSS, the variance contribution of the three factors was estimated using mGENOVA. The results are shown in Table 10. The actual variance contributions of F1, F2, and F3 were 24.67%, 21.85%, and 53.48%, respectively, which closely aligned with the proportions of items assigned to each dimension (F1 = 27.78%, F2 = 22.22%, F3 = 50%). This consistency indicates that the contribution of each factor to the total score is proportionate to its item allocation, supporting the use of the HSSS for both norm-referenced and criterion-referenced assessment.

**Table 10:** Comparison of variance contributions and item proportions across the three factors.

Variance contribution	F1	F2	F3
Total score for the whole domain	24.67%	21.85%	53.48%
Relative error	32.20%	18.21%	49.59%
Absolute error	32.11%	18.65%	49.24%
Number of items for each factor	5	4	9
Total score of each factor	25	20	45
Proportion of scores for each factor	27.78%	22.22%	50%

Effect of the number of items per dimension on measurement accuracy. To examine how measurement precision changes under different measurement conditions, the number of items per dimension was systematically varied in the D-study. As shown in Table 11, when each dimension contained only one item, the generalization coefficient of the total score decreased to 0.751, which is below the acceptable threshold. When the number of items per dimension was doubled (i.e., 10, 8, and 18 items for F1, F2, and F3, respectively), the generalization coefficient increased to 0.973; when tripled (i.e., 15, 12, and 27 items), it further increased to 0.982. These results indicate that increasing the number of items per dimension improves measurement precision, consistent with classical measurement theory. The improvement showed diminishing returns: the gain from the baseline model to the 2-fold model was 0.222, whereas the gain from the 2-fold model to the 3-fold model was only 0.009. When the number of items for only one dimension was varied while holding the other two constant, the generalization coefficient improved, but the magnitude of improvement differed across dimensions. The current item allocation (5, 4, and 9 items for F1, F2, and F3) thus provides a reasonable balance between measurement precision and scale length.

**Table 11:** Relationship between sample size and generalizability coefficient for total score for each dimension in the D-study.

D-study design	$n_{1}^{\prime }$	$n_{2}^{\prime }$	$n_{3}^{\prime }$	Generalizability coefficient for total score	Changes in the generalizability coefficient
D Research Variables	5	4	9	0.948	
Baseline model	1	1	1	0.751	
2-fold model	10	8	18	0.973	+0.222
3-fold model	15	12	27	0.982	+0.009
Only $n_{1}^{\prime }$ change	1	4	9	0.942	
	10	4	9	0.957	+0.015
	15	4	9	0.963	+0.006
Only $n_{2}^{\prime }$ change	5	1	9	0.938	
	5	8	9	0.959	+0.021
	5	12	9	0.966	+0.007
Only $n_{3}^{\prime }$ change	5	4	1	0.909	
	5	4	18	0.967	+0.058
	5	4	27	0.976	+0.009

### 3.6 Composite reliability analysis

The HSSS comprises three sub-dimensions, and composite reliability (CR) was calculated for each dimension following Sijtsma (2009). The CR values ranged from 0.838 to 0.923, all exceeding the recommended criterion of 0.70, which indicates acceptable internal consistency for each factor. In addition, most item-level reliability estimates fell within the acceptable range, suggesting that each item reasonably represents its intended dimension. Detailed results are presented in Table 12.

**Table 12:** Composite reliability and item-level reliability of the HSSS.

Path			Parameter significant type estimation project credibility				Combinatorial reliability
			Estimate	S.E.	*t*-value	SMC	CR
S3	←	F1	0.661			0.437	
S6	←	F1	0.696	0.086	12.156	0.484	
S8	←	F1	0.754	0.081	12.949	0.569	0.838
S9	←	F1	0.765	0.084	13.071	0.585	
S10	←	F1	0.689	0.086	11.954	0.475	
S18	←	F2	0.799			0.638	
S19	←	F2	0.816	0.055	17.619	0.666	0.861
S20	←	F2	0.828	0.056	17.949	0.686	
S22	←	F2	0.670	0.055	14.119	0.449	
S26	←	F3	0.680			0.462	
S28	←	F3	0.700	0.071	13.467	0.490	
S29	←	F3	0.732	0.073	13.907	0.536	0.923
S30	←	F3	0.769	0.074	14.594	0.591	
S31	←	F3	0.730	0.077	13.887	0.533	
S32	←	F3	0.824	0.079	15.344	0.679	
S33	←	F3	0.802	0.076	14.959	0.643	
S34	←	F3	0.783	0.081	14.616	0.613	
S35	←	F3	0.777	0.077	14.590	0.604	

*N* = 427. F1 = Withdrawal from avoidant speech and behavior; F2 = Negative self-consciousness; F3 = Negative emotional experience.

### 3.7 Internal consistency and test–retest reliability

Internal consistency and test–retest reliability were examined for the HSSS. The Cronbach’s *α* coefficient of the total scale was 0.935, and the Cronbach’s *α* coefficients for the three dimensions ranged from 0.836 to 0.923 (F1 = 0.836, F2 = 0.859, F3 = 0.923). The test–retest reliability of the total scale was 0.894, and the test–retest reliabilities for the three dimensions were 0.727 (F1), 0.724 (F2), and 0.763 (F3), all of which fell within the acceptable range. These results indicate that the HSSS demonstrated acceptable internal consistency and temporal stability.

### 3.8 Content validity

To evaluate the content validity of the HSSS, nine professional psychologists were invited to conduct item evaluation and screening. The results showed that the item-level content validity index (I-CVI) of all items was above 0.78, and the corresponding K^*^ value exceeded 0.74, meeting the psychometric criteria for acceptable item content validity (Shi et al., 2012). The average scale-level content validity index (S-CVI/Ave) was 0.91, exceeding the recommended cutoff of 0.90 (Waltz et al., 2010). These results indicate that the HSSS exhibits acceptable content validity, with all items covering the core conceptual connotation of shyness among Chinese high school students. Detailed results are presented in Table 13.

**Table 13:** Calculation of content validity expert ratings and content validity index for the shyness scale for high school students.

Term	Specialists									Experts with a rating of 3 or 4	I-CVI	pc	*k* ^*^	Estimation
	A	B	C	D	E	F	G	H	I					
1	3	4	4	3	4	3	4	3	2	8	0.89	0.018	0.89	Excellence
2	4	4	4	4	3	4	4	3	3	9	1.00	0.002	1.00	Excellence
3	3	4	4	2	3	3	4	4	2	7	0.78	0.070	0.76	Excellence
4	3	3	4	3	3	4	4	4	3	9	1.00	0.002	1.00	Excellence
5	3	4	4	4	3	4	4	4	2	8	0.89	0.018	0.89	Excellence
6	3	2	4	4	3	4	4	3	4	8	0.89	0.018	0.89	Excellence
7	4	4	4	4	4	4	4	4	4	9	1.00	0.002	1.00	Excellence
8	4	4	3	4	4	3	3	3	4	9	1.00	0.002	1.00	Excellence
9	3	4	3	4	3	4	4	3	4	9	1.00	0.002	1.00	Excellence
10	4	3	4	3	4	4	4	4	4	9	1.00	0.002	1.00	Excellence
11	3	4	2	4	3	3	3	3	2	7	0.78	0.070	0.76	Excellence
12	4	3	3	4	2	4	3	4	3	8	0.89	0.018	0.89	Excellence
13	3	2	3	2	3	3	4	3	3	7	0.78	0.002	0.76	Excellence
14	3	4	4	4	4	4	4	4	4	9	1.00	0.002	1.00	Excellence
15	4	4	3	4	4	3	4	2	4	8	0.89	0.018	0.89	Excellence
16	4	3	3	4	3	4	3	4	3	9	1.00	0.002	1.00	Excellence
17	2	3	4	2	3	4	4	3	3	7	0.78	0.070	0.76	Excellence
18	4	4	2	4	4	2	4	3	4	7	0.78	0.070	0.76	Excellence

I-CVI = item-level content validity index; S-CVI/Ave = average scale-level content validity index; k* = modified kappa, which adjusts the I-CVI for the possibility of random agreement among experts. The formula is k* = (I-CVI - pc) / (1 - pc), where pc is the probability of random agreement. Values above 0.74 are considered excellent.

### 3.9 Convergent validity

Convergent validity refers to the extent to which items measuring the same construct are highly correlated and load on the same factor (Sijtsma, 2009). For the HSSS, the average variance extracted (AVE) values for the three dimensions ranged from 0.510 to 0.610 (F1 = 0.510, F2 = 0.610, F3 = 0.572), all exceeding the recommended criterion of 0.50. These results indicate that each dimension demonstrates acceptable convergent validity and explains a sufficient proportion of variance in its indicators. Detailed results are presented in Table 14.

**Table 14:** Convergent validity of the HSSS.

Path			Estimate	AVE
S3	←	F1	0.661	
S6	←	F1	0.696	
S8	←	F1	0.754	0.510
S9	←	F1	0.765	
S10	←	F1	0.689	
S18	←	F2	0.799	
S19	←	F2	0.816	0.610
S20	←	F2	0.828	
S22	←	F2	0.670	
S26	←	F3	0.680	
S28	←	F3	0.700	
S29	←	F3	0.732	0.572
S30	←	F3	0.769	
S31	←	F3	0.730	
S32	←	F3	0.824	
S33	←	F3	0.802	
S34	←	F3	0.783	
S35	←	F3	0.777	

*N* = 427. F1 = Withdrawal from avoidant speech and behavior; F2 = Negative self-consciousness; F3 = Negative emotional experience.

### 3.10 Discriminant validity

Discriminant validity refers to the extent to which a construct is empirically distinct from other constructs. For the HSSS, discriminant validity was examined using the Fornell-Larcker criterion, which requires that the square root of the average variance extracted (AVE) for each dimension be greater than its correlations with the other dimensions (Fornell and Larcker, 1981; Pieters et al., 2026). As shown in Table 15, the square roots of AVE (bolded on the diagonal) ranged from 0.714 to 0.781, all of which exceeded the corresponding inter-dimension correlations (0.618–0.748). These results indicate that each dimension shares more variance with its own indicators than with other dimensions, supporting the discriminant validity of the HSSS.

**Table 15:** Discriminant validity of the High School Student Shyness Scale.

DIM	AVE	F1	F2	F3
F1	0.510	**0.714**		
F2	0.610	0.691	**0.781**	
F3	0.572	0.618	0.748	**0.756**

Bolded numbers on the diagonal are the square root of the average variance extracted (√AVE) for the corresponding dimension; off-diagonal numbers are the correlation coefficients between dimensions. F1 = Withdrawal from avoidant speech and behavior; F2 = Negative self-consciousness; F3 = Negative emotional experience.

### 3.11 Measurement invariance across gender

To examine whether the HSSS measures the same construct across gender, a series of measurement invariance tests were conducted using multi-group CFA. As shown in Table 16, the unconstrained (configural) model showed acceptable fit (*χ*^2^ = 576.641, *df* = 264, CFI = 0.930, TLI = 0.919, RMSEA = 0.053). The metric model, which constrained all factor loadings to be equal across groups, also showed acceptable fit (*χ*^2^ = 596.727, *df* = 279, CFI = 0.929, TLI = 0.922, RMSEA = 0.052), with ΔCFI = 0.001 and ΔRMSEA = 0.001. The scalar model, which further constrained all item intercepts to be equal, showed acceptable fit (*χ*^2^ = 625.194, *df* = 297, CFI = 0.927, TLI = 0.925, RMSEA = 0.051), with ΔCFI = 0.002 and ΔRMSEA = 0.001. The strict model, which constrained error variances to be equal, showed acceptable fit (*χ*^2^ = 654.662, *df* = 321, CFI = 0.926, TLI = 0.929, RMSEA = 0.049), with ΔCFI = 0.001 and ΔRMSEA = 0.002. All ΔCFI values were below the recommended cutoff of 0.01, and all ΔRMSEA values were below 0.015, indicating that measurement invariance across gender was supported. These results suggest that the HSSS measures shyness equivalently for male and female high school students.

**Table 16:** Measurement invariance across gender.

Model	*χ* ^2^	*df*	*χ*^2^/*df*	CFI	TLI	RMSEA	ΔCFI	ΔRMSEA
Unconstrained (Configural)	576.641	264	2.184	0.930	0.919	0.053	–	–
Metric	596.727	279	2.139	0.929	0.922	0.052	0.001	0.001
Scalar	625.194	297	2.105	0.927	0.925	0.051	0.002	0.001
Strict	654.662	321	2.039	0.926	0.929	0.049	0.001	0.002

*N* = 427 (Male = 172, Female = 255). All models were estimated using maximum likelihood in Amos 26.0. ΔCFI < 0.01 and ΔRMSEA < 0.015 indicate support for invariance (Cheung and Rensvold, 2002).

### 3.12 Measurement invariance across grade level

To examine whether the HSSS measures the same construct across grade levels, a series of measurement invariance tests were conducted using multi-group CFA. As shown in Table 17, the unconstrained (configural) model showed acceptable fit (*χ*^2^ = 746.622, *df* = 396, CFI = 0.924, TLI = 0.911, RMSEA = 0.046). The metric model, which constrained all factor loadings to be equal across groups, also showed acceptable fit (*χ*^2^ = 788.643, *df* = 426, CFI = 0.921, TLI = 0.915, RMSEA = 0.045), with ΔCFI = 0.003 and ΔRMSEA = 0.001. The scalar model, which further constrained all item intercepts to be equal, showed acceptable fit (*χ*^2^ = 836.736, *df* = 462, CFI = 0.918, TLI = 0.919, RMSEA = 0.044), with ΔCFI = 0.003 and ΔRMSEA = 0.001. The strict model, which constrained error variances to be equal, showed acceptable fit (*χ*^2^ = 932.696, *df* = 510, CFI = 0.908, TLI = 0.917, RMSEA = 0.044), with ΔCFI = 0.010 and ΔRMSEA = 0.000. The ΔCFI value was at the recommended cutoff of 0.01, and the ΔRMSEA value was below 0.015, indicating that strict measurement invariance across grade level was partially supported. These results suggest that the HSSS measures shyness equivalently across grade levels.

**Table 17:** Measurement invariance across grade level.

Model	*χ* ^2^	*df*	*χ*^2^/*df*	CFI	TLI	RMSEA	ΔCFI	ΔRMSEA
Unconstrained (configural)	746.622	396	1.885	0.924	0.911	0.046	–	–
Metric	788.643	426	1.851	0.921	0.915	0.045	0.003	0.001
Scalar	836.736	462	1.811	0.918	0.919	0.044	0.003	0.001
Strict	932.696	510	1.829	0.908	0.917	0.044	0.010	0.000

N = 427 (Grade 10 = 190, Grade 11 = 132, Grade 12 = 105). All models were estimated using maximum likelihood in Amos 26.0. ΔCFI < 0.01 and ΔRMSEA < 0.015 indicate support for invariance (Cheung and Rensvold, 2002).

### 3.13 Structural validity

Structural validity was examined by correlating the total score with each dimension and by examining the inter-dimension correlations. As shown in Table 18, the correlations between the total score and the three dimensions ranged from 0.787 to 0.926, indicating that each dimension was moderately to highly correlated with the total score. The correlations among the three dimensions ranged from 0.547 to 0.681, indicating that the dimensions were moderately correlated but remained sufficiently independent from one another. These results support the structural validity of the HSSS.

**Table 18:** Structural validity of the HSSS.

Variability	F1	F2	F3
Withdrawal from avoidant speech and behavior	1		
Negative self-consciousness	0.594^**^	1	
Negative emotional experiences	0.547^**^	0.681^**^	1
total score	0.787^**^	0.839^**^	0.926^**^

^**^p < 0.01. F1 = Withdrawal from avoidant speech and behavior; F2 = Negative self-consciousness; F3 = Negative emotional experience.

### 3.14 Criterion-related validity

To examine the criterion-related validity of the HSSS, the Cheek and Buss Shyness Scale was used as the criterion measure and administered to a separate sample of 200 high school students (Sample 5). The Cheek and Buss Shyness Scale is one of the most widely used measures of global shyness in both Western and Chinese samples (Cheek and Buss, 1981; Crozier, 2005), which makes it a well-established criterion for assessing convergent validity. According to the framework of construct validity proposed by Campbell and Fiske (1959), evidence of convergent validity emerges when different measures assessing the same trait yield consistent results. Therefore, a significant correlation between a unidimensional measure of global shyness (the Cheek and Buss Shyness Scale) and a multidimensional measure (the HSSS) would provide evidence of convergent validity for the HSSS, as both instruments are intended to assess the overarching construct of shyness. The Cronbach’s *α* of the Cheek and Buss Shyness Scale in this sample was 0.778, indicating acceptable internal consistency. Pearson product–moment correlation analysis was conducted to examine the relationship between the total score of the HSSS and the total score of the Cheek and Buss Shyness Scale. The results showed a significant positive correlation between the two measures, *r* = 0.771, *p* < 0.001, indicating that the HSSS demonstrates acceptable criterion-related validity and can reasonably measure shyness levels in Chinese high school students.

## 4 Discussion

### 4.1 Cultural and theoretical foundations of the HSSS

Drawing on mature theoretical systems and abundant empirical findings in international shyness research, High School Student Shyness Scale (HSSS) was developed and psychometrically validated within China’s indigenous cultural context and the developmental characteristics of senior high school students. A stable three-factor structure was identified: Withdrawal from avoidant speech and behavior, Negative self-consciousness, and Negative emotional experience. All psychometric indicators generally conform to the criteria of standardized measurement tools, suggesting that the HSSS possesses a stable structure and acceptable measurement accuracy for assessing shyness among Chinese senior high school students.

Traditional Chinese culture has long advocated modesty, restraint, and gentleness, subtly shaping reserved and low-key social behavioral tendencies and enabling introverted social patterns to gain high acceptance in the local cultural context. Contemporary social changes have gradually reshaped public cognition and evaluation of shyness, which is reflected in the present data. Chinese senior high school students hold predominantly negative views of shyness and can capture its adverse effects at cognitive, emotional, and behavioral levels, especially its obstructive impact on social adaptability. This feature is consistent with mainstream international findings, supporting the cross-cultural consistency of shyness as a stable personality trait. This consistency, however, does not negate culturally specific manifestations.

The social adaptation model of shyness (Henderson et al., 2010) provides a coherent framework for understanding the occurrence and development of shyness among Chinese senior high school students. In the complete social chain of social approach, real-time interaction, and post-social evaluation, shy individuals automatically activate negative social expectations, which gradually solidify into stable negative thinking patterns and trigger a series of adaptive problems, including physical and mental discomfort, emotional suppression, and behavioral inhibition. Students with high shyness levels are generally sensitive, psychologically vulnerable, low in self-identity, and persistently depressed in emotions, making them prone to developmental deficiencies such as insufficient social competence and emotional dysregulation (Brunetti et al., 2024; Sequeira et al., 2025). Long-term accumulation of these unresolved problems may further lead to psychological and social maladjustment, exerting a significant negative impact on individuals’ physical and mental health and long-term personality development (Kim and Rajaguru, 2025).

### 4.2 Multidimensional structure and its theoretical implications

The three-dimensional structure established in this study corresponds to the core cognitive-affective-behavioral framework of shyness, with each dimension supported by theoretical and empirical evidence. Negative self-consciousness serves as the core cognitive kernel of shyness, aligning with the developmental rules of adolescent ego identity. According to Erikson's (1968) ego identity theory, the core developmental task of adolescence is to construct an independent, stable, and integrated self-cognitive system. Shy individuals tend to be more concerned about others’ evaluations and to report lower self-esteem (Koydemir-Özden and Demir, 2009; Wei et al., 2015). This pattern may also be relevant to Chinese senior high school students, who tend to rely on external evaluation feedback and lack stable self-worth recognition. Long-term negative cognitive states may lead to ego identity diffusion or negative identity deviation and hinder the cultivation of a healthy personality, validating Crozier's (1979) cognitive theory of shyness. Shy individuals generally exhibit negative self-schemas and negative self-evaluation tendencies, accompanied by prominent social avoidance and social maladjustment, supporting the intrinsic cognitive mechanism of shyness (Crozier, 1979; Xiang et al., 2018).

Beyond this cognitive core, the Withdrawal from avoidant speech and behavior dimension directly reflects typical social avoidance and behavioral inhibition traits, consistent with existing international conclusions (Jung et al., 2025; Jung et al., 2026). When facing unfamiliar or high-pressure social situations, shy senior high school students form negative social predictions in advance and overly concern themselves with external evaluations, thereby actively avoiding interpersonal interaction and reducing social participation, which ultimately aggravates social adaptation difficulties (Woody et al., 2022; Leung et al., 2025). This behavioral mechanism is consistent with the social anxiety cognitive behavioral model (Rapee and Heimberg, 1997), indicating that negative social cognition directly induces negative emotions such as social fear and anxiety, and further leads to persistent social avoidance.

Corresponding to this behavioral pattern, the Negative emotional experience dimension covers negative emotional responses throughout pre-social, in-social, and post-social interaction, mainly manifested as persistent tension, panic, anxiety, and restlessness. This feature is consistent with previous indigenous research in China (Wu, 2012). Shy individuals tend to repeatedly review social scenarios, overemphasize their own shortcomings and negative performances, and thus generate stronger and more lasting negative emotions. Risk factors such as social evaluation fear and social rejection expectation further aggravate negative emotional experiences, prompting individuals to avoid social interaction and ultimately forming stable and intractable shyness states (Clifford et al., 2022; Vagos et al., 2025).

These three dimensions are not independent; rather, they are closely interrelated. Negative self-consciousness serves as the cognitive basis for negative emotions and behavioral withdrawal; Negative emotional experience reciprocally strengthens negative cognition and induces avoidant behaviors; and withdrawal behaviors further solidify maladaptive cognition and negative emotions. This mutually reinforcing dynamic shapes the cognitive resource allocation, behavioral tendencies, and emotional states of shy senior high school students, jointly explaining the occurrence and development of shyness among Chinese senior high school students.

Although the three dimensions are theoretically and empirically interrelated, it remains to be examined whether they are better represented by a single general factor or by distinct specific factors. The bifactor indices indicated that the general factor accounted for the majority of common variance and total score reliability, whereas the specific factors provided limited reliable information. Together with the high inter-factor correlations, this pattern suggests that most of the reliable variance is attributable to the general factor. Accordingly, the total score may be recommended as the primary indicator of shyness, and subscale scores, particularly F3, should be interpreted with caution. The first-order three-factor model is retained as the most parsimonious and interpretable measurement model.

### 4.3 Cultural adaptation and innovative contributions

Although the three-factor structure of the HSSS conceptually aligns with universal components of shyness identified in Western literature, we argue that the instrument is culturally adapted rather than culturally specific, as its dimensions may be shaped by the Chinese high school context. Negative self-consciousness may reflect the Confucian emphasis on shame and “face” (mianzi), where self-doubt is triggered not merely by individual performance failure, but by the fear of failing to meet collective expectations (Ho, 1976; Hwang, 1987); items such as S18 may capture this dimension. Withdrawal from avoidant speech and behavior may be culturally nuanced: under the influence of the “Doctrine of the Mean,” Chinese adolescents may withdraw to maintain interpersonal harmony, rather than solely to avoid anxiety. Items such as S6 may reflect this cultural nuance. Negative emotional experience may be exacerbated by the increasingly competitive academic environment of Chinese senior high schools, in which shyness is less compatible with the emphasis on active social engagement (Chen et al., 2005). Items such as S32 may capture this cultural aspect. Therefore, while the HSSS shares universal structural elements with existing measures, it may capture specific contextual and cultural nuances relevant to assessing Chinese high school students.

### 4.4 Comparison with existing Chinese shyness scales

The HSSS represents one of the first psychometrically validated, multidimensional shyness scales specifically designed for Chinese senior high school students. To clarify its contribution, we compare its dimensional structure with two existing indigenous Chinese shyness scales: the College Student Shyness Scale (Wang, 2007) and the Junior High School Student Shyness Scale (Chen, 2013).

The College Student Shyness Scale identified four dimensions: seeking approval, self-blame, fear of rejection, and self-restriction in expression. While these dimensions partially overlap with the HSSS’s cognitive dimension (Negative self-consciousness), the college-oriented scale focuses primarily on cognitive and emotional aspects and lacks a distinct behavioral withdrawal dimension. In contrast, the HSSS incorporates Withdrawal from avoidant speech and behavior as a separate dimension, capturing behavioral manifestations particularly salient in high school campus settings (e.g., avoiding public speaking, staying quiet in class). In addition, the college-oriented scale assumes a level of social maturity that may not yet be fully developed in high schoolers, whereas the HSSS is tailored to the developmental stage of adolescence.

The Junior High School Student Shyness Scale identified dimensions such as modesty shyness and heterosocial shyness. However, these dimensions are embedded in adolescent social contexts that may not fully capture the complex social situations of senior high school students (e.g., public speaking, opposite-sex interactions, peer evaluation; Chen, 2013). The HSSS, by contrast, is grounded in the social adaptation model of shyness and covers the complete social interaction cycle, making it more developmentally appropriate for adolescents.

Compared with these two existing indigenous scales, the HSSS contributes to the Chinese shyness measurement literature in three ways. First, it addresses a developmental gap by providing a psychometrically validated, multidimensional shyness scale specifically designed for Chinese senior high school students—a population not fully served by child-oriented or college-oriented scales. Second, it integrates cognitive, affective, and behavioral dimensions within a single multidimensional instrument, offering a more comprehensive assessment than unidimensional scales (Cheek and Buss, 1981). Third, it is culturally adapted to the Chinese high school context while remaining theoretically grounded in international shyness research, thereby balancing cultural sensitivity with cross-cultural comparability.

### 4.5 Contribution of multivariate generalizability theory

In the single-facet *p* × *i* design, the generalizability coefficients for the three dimensions are mathematically equivalent to Cronbach’s *α*. This equivalence does not diminish the value of MGT for the present study. MGT extends conventional reliability estimation in three substantive ways: it decomposes measurement error into participant, item, and participant × item interaction components, thereby identifying the specific sources of measurement error; it permits D-studies to evaluate how measurement precision would change under alternative measurement designs, such as increasing the number of items per dimension; and it allows the simultaneous estimation of measurement precision and inter-dimension correlations across the three dimensions, which is not possible with Cronbach’s *α*. These features make MGT particularly informative for evaluating the measurement precision of multidimensional instruments such as the HSSS.

### 4.6 Limitations and future directions

#### 4.6.1 Factor-analytic procedures

The EFA was conducted using Principal Axis Factoring (PAF) with Promax oblique rotation and parallel analysis. PAF was selected over PCA because it is a common factor model suited to identifying latent constructs; Promax was chosen because the three shyness dimensions were expected to be correlated; and parallel analysis was used to determine the number of factors to retain. This combination supported the robustness of the three-factor structure.

Two methodological limitations warrant discussion. First, the qualitative responses were coded deductively against the pre-established three-dimensional model. The absence of newly emerging dimensions was therefore expected by design and provides limited independent support for the three-factor structure. Second, the data-driven item deletion during CFA may risk capitalization on chance. Two of the three items removed during CFA (Items 15 and 23) belonged to the Negative self-consciousness dimension (F2), which now consists of only four items. Although these four items demonstrated acceptable internal consistency (Cronbach’s *α* = 0.861) and the final model showed good fit, the relatively small number of items in F2 may limit its content coverage.

Future research could address these limitations by using inductive or mixed-methods approaches to further validate the dimensional structure of the HSSS, examining whether additional items could strengthen the Negative self-consciousness dimension, and testing measurement invariance across regions and demographics.

#### 4.6.2 Bifactor model

The bifactor model was examined only in the present sample. Future research could further evaluate the bifactor model with larger and more diverse samples, which would help clarify the practical contribution of the general and specific factors.

#### 4.6.3 Validity evidence

The present study provided evidence for convergent and criterion-related validity. Several aspects of validity remain to be addressed. As the current dataset did not include measures of related but distinct constructs (e.g., social anxiety, introversion), discriminant validity relative to these constructs was not assessed. The dataset also lacked outcome variables such as loneliness or academic adjustment; consequently, predictive and incremental validity could not be evaluated. Given the multidimensional structure of the HSSS, incorporating relevant comparison measures and outcome variables in future research would help establish a more comprehensive nomological network.

A further consideration concerns the cross-validation of the 18-item model. Sample 5 (*N =* 200) was originally collected for criterion-related validity and differs from Sample 3 in both size and sampling procedure. The cross-validation yielded acceptable *χ*^2^/*df* but somewhat lower CFI and TLI, and a higher RMSEA, than those observed in Sample 3. These findings suggest that the discrepancy in fit may reflect differences in sample characteristics rather than a substantive weakness of the factor structure. Future research should cross-validate the 18-item model in larger and more representative samples.

#### 4.6.4 Measurement invariance

Measurement invariance was tested across gender and grade level at the configural, metric, scalar, and strict levels. The strict invariance model for grade level showed a ∆CFI of 0.010, which is at the recommended cutoff of 0.01 (Cheung and Rensvold, 2002). This suggests that strict invariance across grade level was partially supported, and the finding should be interpreted with appropriate caution. In contrast, strict invariance across gender was fully supported (∆CFI = 0.001). Future research with more diverse samples could further examine whether these invariance patterns hold across other demographic and regional subgroups.

#### 4.6.5 Cultural hypotheses and cross-cultural generalizability

The cultural interpretations proposed in the present study, such as the roles of mianzi and the “Doctrine of the Mean,” are better regarded as plausible cultural hypotheses rather than empirically established characteristics of the HSSS. Future research could directly test whether these cultural factors shape the expression of shyness and whether the corresponding items function differently across cultural contexts. Cross-cultural studies may also benefit from examining measurement invariance across diverse cultural groups, so as to further disentangle universal versus culture-specific aspects of shyness. Such investigations may, in turn, encourage a re-examination of previous cross-cultural comparisons of shyness, which often relied on unidimensional measures, using multidimensional instruments such as the HSSS.

#### 4.6.6 Sample representativeness and cross-regional invariance

All samples were drawn from district and county-level high schools in Chongqing, China, using convenience sampling. Students in these schools came from both urban and rural household registration backgrounds; however, all participants were drawn from Chongqing, and no participants from other provinces were included. Consequently, the sample may not fully represent high school students from other provinces or regions with different socioeconomic and cultural backgrounds. Furthermore, measurement invariance across different economic and cultural regions was not tested, leaving it unknown whether the three-factor structure of the HSSS is invariant across diverse regional backgrounds. Previous research has documented significant regional differences in shyness and social withdrawal among Chinese adolescents (Chen et al., 2005); therefore, the generalizability of the present findings to high school students in other provinces or regions of China requires future examination. Finally, the reliance on convenience sampling may introduce selection bias. Future research should adopt multi-regional, stratified random sampling and conduct cross-regional measurement invariance tests to verify the applicability and structural stability of the HSSS across diverse Chinese high school populations.

## 5 Conclusion

The present study developed and validated the High School Student Shyness Scale (HSSS), an 18-item instrument comprising three dimensions: Withdrawal from avoidant speech and behavior, Negative self-consciousness, and Negative emotional experience. The HSSS demonstrated acceptable psychometric properties, including evidence of structural validity, high measurement precision, and measurement invariance across gender and grade level. The core three-factor structure was broadly replicated in an independent sample, although the fit was somewhat less optimal than in the primary sample, suggesting that the structure is broadly replicable but warrants further validation. These findings suggest that the HSSS is a culturally adapted tool suitable for assessing shyness among Chinese high school students.

## Data Availability

The raw data supporting the conclusions of this article will be made available by the authors, without undue reservation.
