# Supplementary material for: Development of a shyness scale for Chinese high school students
Source: Front Psychol. 2026 Oct 22;17:1895425. doi: 10.3389/fpsyg.2026.1895425 (PMC13736733; doi:10.3389/fpsyg.2026.1895425)
Supplement: Supplementary file 1 [file Supplementary_file_1.pdf]

APPENDIX A

High School Student Shyness Scale (HSSS): Development of the Preliminary Scale

Item (English)	Initial Item (Chinese)	strongly disagree	disagree	uncertain	agree	strongly agree
S1. In a crowded place, I always hide where no one can notice me.	在人多的地方，我总是躲到没人注意我的角落。					
S2. I will try to avoid situations where I might feel embarrassed.	我会尽量避免那些可能让我感到尴尬的场合。					
S3. When talking to people I don't know well, I always find an excuse to leave.	与不太熟悉的人交谈时，我总是找借口离开。					
S4. I will avoid being the center of attention in social situations.	在社交场合中，我会避免成为大家关注的焦点。					
S5. I always keep my head down in embarrassment when I meet with others.	与人见面时，我总是因为尴尬而低着头。					
S6. I try to avoid situations that force me to socialize.	我尽量避免那些不得不社交的场合。					
S7. I am often afraid to speak my inner truth.	我常常不敢说出自己的真实想法。					
S8. In social situations, I feel restricted in my speech and behavior.	在社交场合中，我觉得自己的言行受到拘束。					
S9. In public, I get very quiet when I talk.	在公共场合说话时，我会变得沉默					

	寡言。					
S10. When facing many people looking at me, I will keep doing some small actions to cover up (e.g. biting my fingers, touching my head, etc.).	面对很多人注视时，我会不停做一些小动作来掩饰（如咬手指、摸头等）。					
S11. When talking with a classmate or friend, I don't dare to look them in the eyes.	与同学或朋友交谈时，我不敢直视对方的眼睛。					
S12. In social situations, I am often so nervous that I blush.	在社交场合中，我常常紧张到脸红。					
S13. I always feel bad about myself in public or unfamiliar situations.	在公共场合或不熟悉的场合中，我总是觉得自己不够好。					
S14. I always consider myself to be an embarrassment at social events.	我总觉得自己在社交活动中是个令人尴尬的人。					
S15. I often feel that people look down on me.	我常常觉得别人看不起我。					
S16. I often think I do or say the wrong thing in social situations.	在社交场合中，我常常觉得自己说错话或做错事。					
S17. I always feel like I look funny to others.	我总是觉得在别人眼里自己很可笑					
S18. I have always found myself unable to meet the expectations of others from time to time.	我时常发现自己无法达到别人的期望。					
S19. From time to time, I think I am	我时常觉得					

a failure in interpersonal relationships.	自己在人际关系中是个失败者。					
S20. I always suspect that I am incapable.	我总是怀疑自己能力不足。					
S21. I always feel that others think I am not good enough.	我总觉得别人认为我不够好。					
S22. In interpersonal relationships, I always feel that people do not like or accept me.	在人际关系中，我总觉得别人不喜欢我或不接纳我。					
S23. I always feel that I don't have enough self-confidence to speak at parties or other occasions.	我总觉得在聚会或其他场合发言时缺乏自信。					
S24. I often feel that I make a bad impression on others.	我常常觉得自己给别人留下了不好的印象。					
S25. In social situations, I am always afraid that people will laugh at me.	在社交场合中，我总是害怕别人会嘲笑我。					
S26. When I think about talking to people, I always worry about what they will think of me.	一想到要与人交谈，我就担心他们会怎么看我。					
S27. I'm always afraid that people won't think I'm good enough.	我总是害怕别人觉得我不够好。					
S28. I begin to feel nervous at the thought of meeting and communicating with others.	一想到要与人见面和交流，我就开始感到紧张。					
S29. I feel scared when people make negative comments about me in interpersonal situations.	在人际情境中，当别人对我作出负面评价时,					

	我会感到害怕。					
S30. I often feel isolated and alienated in social situations.	我常在社交场合中感到被孤立和疏远。					
S31. I feel awkward in social situations when everyone is silent.	当大家沉默时，我在社交场合中感到尴尬。					
S32. I often feel ashamed of myself for avoiding socializing.	我常因回避社交而对自己感到羞愧。					
S33. After socializing, I often feel self-conscious.	社交之后，我常感到不自在。					
S34. I cringe at my poor performance at social events.	我对自己在社交场合中的糟糕表现感到难堪。					
S35. In crowded situations, I feel panicked when I am noticed by others.	在拥挤的场合中，当我被别人注意到时，我会感到恐慌。					
S36. In social situations, I am always worried about saying or doing the wrong thing.	在社交场合中，我总是担心说错话或做错事。					
S37. When I don't know what to say or do in social situations, I feel nervous.	在社交场合中，当我不知道说什么或做什么时，我会感到紧张。					
S38. I get frustrated when I am criticized by others.	当我被别人批评时，我会感到沮丧					
S39. I feel embarrassed when I disagree with others.	当我与别人意见不同时，我会感到尴尬。					

S40. After socializing, I often feel empty or lost.	社交之后，我常感到空虚或迷茫。					
S41. I wish I were more comfortable in my interactions with others.	我希望自己在与人交往时能更自在。					
S42. I leave social occasions with a sense of relief.	我离开社交场合时会感到如释重负。					

Appendix B

Item Origin, Modification, and Retention Record

Item No.	Initial Item(English)	Initial Item(Chinese)	Originand Modification	Retained in Final18?	Standardized Factor Loading
S1	In a crowded place, I always hide where no one can notice me.	在人多的地方，我总是躲到没人注意我的角落。	Indigenous (open-ended questionnaire)	No	——
S2	I will try to avoid situations where I might feel embarrassed.	我会尽量避免那些可能让我感到尴尬的场合。	Indigenous (open-ended questionnaire)	No	——
S3	When talking to people I don't know well, I always find an excuse to leave.	与不太熟悉的人交谈时，我总是找借口离开。	Indigenous (open-ended questionnaire)	Yes	0.661
S4	I will avoid being the center of attention in social situations.	在社交场合中，我会避免成为大家关注的焦点。	Indigenous (open-ended questionnaire)	No	——
S5	I always keep my head down in embarrassmen	与人见面时，我总是因为尴尬而低着头。	Indigenous (open-ended questionnaire)	No	——

	t when I meet with others.				
S6	I try to avoid situations that force me to socialize.	我尽量避免那些不得不社交的场合。	Indigenous (open-ended questionnaire)	Yes	0.696
S7	I am often afraid to speak my inner truth.	我常常不敢说出自己的真实想法。	Indigenous (open-ended questionnaire)	No	——
S8	In social situations, I feel restricted in my speech and behavior.	在社交场合中，我觉得自己的言行受到拘束。	Indigenous (open-ended questionnaire)	Yes	0.754
S9	In public, I get very quiet when I talk.	在公共场合说话时，我会变得沉默寡言。	Indigenous (open-ended questionnaire)	Yes	0.765
S10	When facing many people looking at me, I will keep doing some small actions to cover up (e.g. biting my fingers, touching my head, etc.).	面对很多人注视时，我会不停做一些小动作来掩饰（如咬手指、摸头等）。	Indigenous (open-ended questionnaire)	Yes	0.689
S11	When talking with a classmate or friend, I don't dare to look them in the	与同学或朋友交谈时，我不敢直视对方的眼睛。	Adapted from Cheek & Buss (1981); simplified to campus	No	——

	eyes.		context		
S12	In social situations, I am often so nervous that I blush.	在社交场合中，我常常紧张到脸红。	Indigenous (open-ended questionnaire)	No	——
S13	I always feel bad about myself in public or unfamiliar situations.	在公共场合或不熟悉的场合中，我总是觉得自己不够好。	Adapted from Cheek & Buss (1981); retained core meaning	No	——
S14	I always consider myself to be an embarrassment at social events.	我总觉得自己在社交活动中是个令人尴尬的人。	Indigenous (open-ended questionnaire)	No	——
S15	I often feel that people look down on me.	我常常觉得别人看不起我。	Indigenous (open-ended questionnaire)	No (deleted in CFA)	——
S16	I often think I do or say the wrong thing in social situations.	在社交场合中，我常常觉得自己说错话或做错事。	Indigenous (open-ended questionnaire)	No	——
S17	I always feel like I look funny to others.	我总是觉得在别人眼里自己很可笑	Indigenous (open-ended questionnaire)	No	——
S18	I have always found myself	我时常发现自己无法达	Indigenous (open-ended	Yes	0.799

	unable to meet the expectations of others from time to time.	到别人的期望。	questionnaire)		
S19	From time to time, I think I am a failure in interpersonal relationships.	我时常觉得自己在人际关系中是个失败者。	Indigenous (open-ended questionnaire)	Yes	0.816
S20	I always suspect that I am incapable.	我总是怀疑自己能力不足。	Indigenous (open-ended questionnaire)	Yes	0.828
S21	I always feel that others think I am not good enough.	我总觉得别人认为我不够好。	Indigenous (open-ended questionnaire)	No	——
S22	In interpersonal relationships, I always think that people don't like me or accept me.	在人际关系中，我总觉得别人不喜欢我或不接纳我。	Indigenous (open-ended questionnaire)	Yes	0.670
S23	I always feel that I don't have enough self-confidence to speak at parties or other occasions.	我总觉得在聚会或其他场合发言时缺乏自信。	Adapted from Cheek & Buss (1981); contextualized to parties	No (deleted in CFA)	——
S24	I often feel that I make a bad	我常常觉得自己给别人留下了不好	Indigenous (open-ended questionnair	No	——

	impression on others.	的印象。	e)		
S25	In social situations, I am always afraid that people will laugh at me.	在社交场合中，我总是害怕别人会嘲笑我。	Indigenous (open-ended questionnaire)	No	——
S26	When I think about talking to people, I always worry about what they will think of me.	一想到要与人交谈，我就担心他们会怎么看我。	Adapted from Cheek & Buss (1981); conceptually aligned	Yes	0.680
S27	I'm always afraid that people won't think I'm good enough.	我总是害怕别人觉得我不够好。	Indigenous (open-ended questionnaire)	No (deleted in CFA)	——
S28	I begin to feel nervous at the thought of meeting and communicating with others.	一想到要与人见面和交流，我就开始感到紧张。	Adapted from Wang (2007); anticipatory anxiety	Yes	0.700
S29	I feel scared when people make negative comments about me in interpersonal situations.	在人际情境中，当别人对我作出负面评价时，我会感到害怕。	Indigenous (open-ended questionnaire)	Yes	0.732
S30	I often feel isolated and alienated in	我常在社交场合中感到被孤立和疏	Indigenous (open-ended questionnair	Yes	0.769

	social situations.	远。	e)		
S31	I feel awkward in social situations when everyone is silent.	当大家沉默时，我在社交场合中感到尴尬。	Indigenous (open-ended questionnair e)	Yes	0.730
S32	I would often feel ashamed of myself for avoiding socializing.	我常因回避社交而对自己感到羞愧。	Adapted from Chen. (2013); shame-relate d item	Yes	0.824
S33	After socializing, I often feel self-conscious .	社交之后，我常感到不自在。	Expert-revis ed (based on open-ended questionnair e and literature)	Yes	0.802
S34	I cringe at my poor performance at social events.	我对自己在社交场合中的糟糕表现感到难堪。	Indigenous (open-ended questionnair e)	Yes	0.783
S35	In crowded situations, I feel panicked when I am noticed by others.	在拥挤的场合中，当我被别人注意到时，我会感到恐慌。	Indigenous (open-ended questionnair e)	Yes	0.777
S36	In social situations, I am always worried about saying or doing the	在社交场合中，我总是担心说错话或做错事。	Indigenous (open-ended questionnair e)	No	——

	wrong thing.				
S37	When I don't know what to say or do in social situations, I feel nervous.	在社交场合中，当我不知道说什么或做什么时，我会感到紧张。	Indigenous (open-ended questionnaire)	No	——
S38	I get frustrated when I am criticized and blamed by others.	当我被别人批评时，我会感到沮丧	Expert-revised (based on open-ended questionnaire and literature)	No	——
S39	I feel embarrassed when I disagree with others.	当我与别人意见不同时，我会感到尴尬。	Expert-revised (based on open-ended questionnaire and literature)	No	——
S40	After socializing, I often feel empty or lost.	社交之后，我常感到空虚或迷茫。	Expert-revised (based on open-ended questionnaire and literature)	No	——
S41	I wish I were more comfortable in my interactions with others.	我希望自己在与人交往时能更自在。	Expert-revised (based on open-ended questionnaire and literature)	No	——
S42	I leave social occasions with a sense of relief.	我离开社交场合时会感到如释重负。	Expert-revised (based on open-ended questionnaire and	No	——

			literature)		

Final 18-Item Composition				
Indigenous (open-ended questionnaire)	14 (77.8%)	3, 6, 8, 9, 10, 18, 19, 20, 22, 29, 30, 31, 34, 35		
Adapted from Cheek & Buss (1981)	1 (5.6%)	26		
Adapted from Wang (2007)	1 (5.6%)	28		
Adapted from Chen (2013)	1 (5.6%)	32		
Expert-revised	1 (5.6%)	33		
Total	18 (100%)			

Note. N=427. Indigenous=items generated from open-ended questionnaire responses collected from 138 high school students. Adapted=items modified from existing standardized shyness scales (Cheek & Buss, 1981; Wang, 2007; Chen, 2013) to fit the Chinese high school context. Expert-revised=items further reviewed and refined by a panel of psychology experts and frontline high school teachers. The adapted items were not adopted wholesale from any single existing scale; rather, they were selected because they reflect core and developmentally appropriate manifestations of shyness that transcend specific measurement contexts. The original scales were not adopted in their entirety due to their conceptual (unidimensional), developmental (child-orcollege-oriented), or cultural (Western or non-high-school) limitations, as detailed in the Introduction. All adapted items were further localized and refined through expert review to ensure relevance to Chinese high school students. "No (deleted in CFA)" refers to items removed during confirmatory factor analysis due to factor loadings below 0.50. Standardized factor loadings are reported for the 18 items retained in the final HSSS (N=427); items not retained in the final scale are marked with "—". All loadings were significant at p<0.001. F1=Withdrawal from avoidant speech and behavior; F2=Negative self-consciousness; F3=Negative emotional experience.
