# Supplementary material for: Development of a shyness scale for Chinese high school students
Source: Front Psychol. 2026 Oct 22;17:1895425. doi: 10.3389/fpsyg.2026.1895425 (PMC13736733; doi:10.3389/fpsyg.2026.1895425)
Supplement: Supplementary file 2 [file Supplementary_file_2.pdf]

Supplementary Material

Supplementary Figure S1

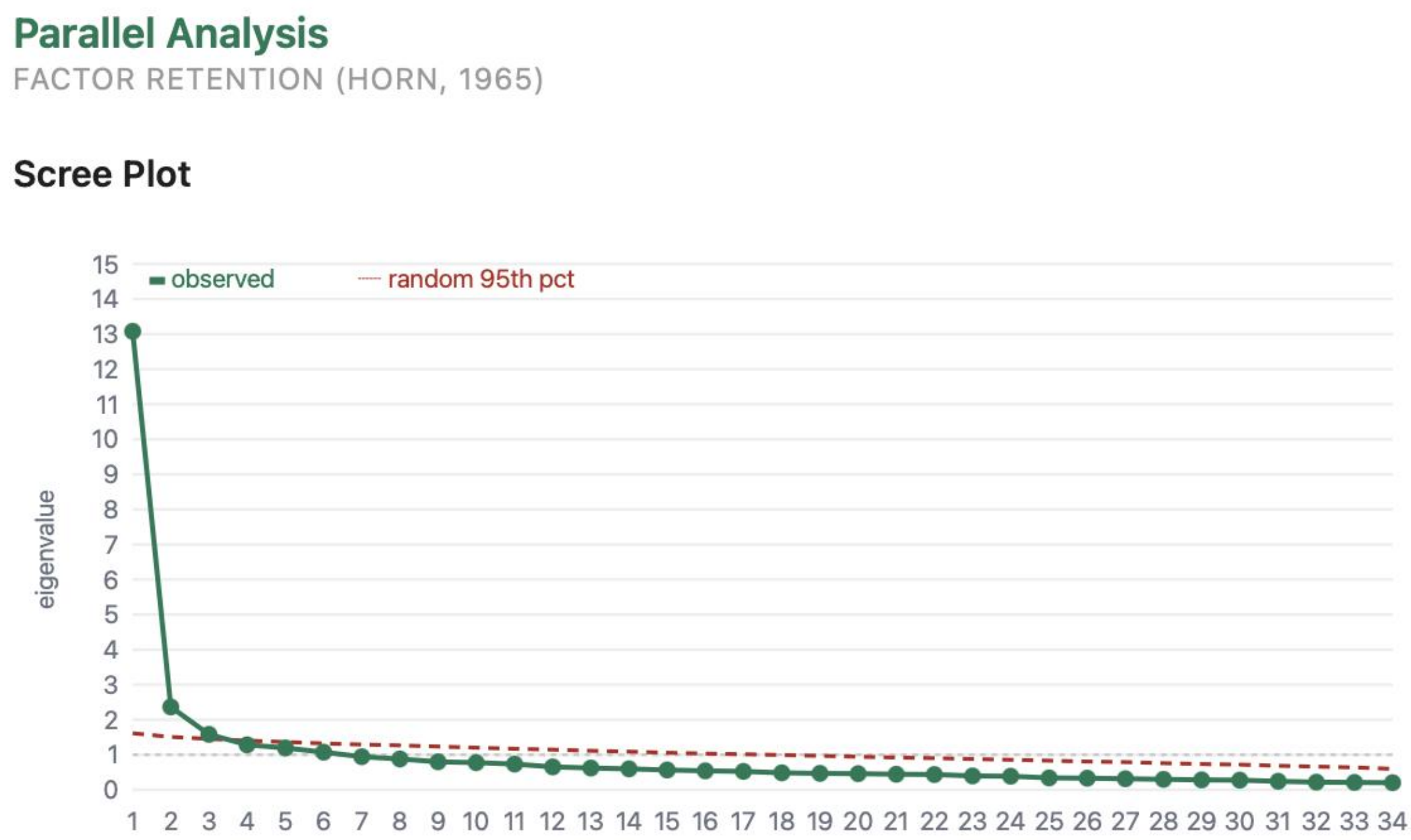

Parallel analysis scree plot of the 34 pre-screened items (*N*=487). The solid green line represents observed eigenvalues from the actual data; the dashed red line represents the 95th percentile of eigenvalues generated from random data. The first three eigenvalues exceed the random threshold, supporting the retention of three factors.

Supplementary Table S1

Parallel Analysis of the 34 Pre-Screened Items

Component	Observed Eigenvalue	Random Mean	Random 95th Percentile	Decision
1	13.080	1.535	1.611	Retain
2	2.366	1.462	1.506	Retain
3	1.583	1.412	1.455	Retain
4	1.285	1.370	1.406	—
5	1.194	1.330	1.364	—
6	1.070	1.294	1.320	—
7	0.940	1.261	1.293	—
8	0.875	1.231	1.267	—
9	0.797	1.203	1.233	—
10	0.777	1.172	1.201	—
11	0.733	1.143	1.167	—
12	0.654	1.116	1.144	—
13	0.618	1.089	1.113	—
14	0.601	1.066	1.090	—
15	0.567	1.039	1.059	—
16	0.538	1.015	1.035	—
17	0.528	0.990	1.015	—
18	0.484	0.966	0.989	—
19	0.471	0.944	0.965	—

Component	Observed Eigenvalue	Random Mean	Random 95th Percentile	Decision
20	0.458	0.922	0.945	—
21	0.446	0.901	0.922	—
22	0.434	0.878	0.899	—
23	0.398	0.855	0.878	—
24	0.385	0.830	0.852	—
25	0.342	0.806	0.827	—
26	0.334	0.784	0.806	—
27	0.315	0.761	0.787	—
28	0.295	0.739	0.761	—
29	0.284	0.715	0.737	—
30	0.276	0.691	0.718	—
31	0.244	0.663	0.686	—
32	0.222	0.638	0.662	—
33	0.211	0.607	0.636	—
34	0.199	0.571	0.597	—

Note. N=487. Random benchmarks were generated from 100 Monte-Carlo samples of uncorrelated normal data of the same size. A component is retained while its observed eigenvalue exceeds the random 95th percentile (Glorfeld, 1995). The first three components satisfied this criterion, supporting the retention of three factors.
